# Strainline: full-length de novo viral haplotype reconstruction from noisy long reads

**DOI:** 10.1186/s13059-021-02587-6

**Published:** 2022-01-20

**Authors:** Xiao Luo, Xiongbin Kang, Alexander Schönhuth

**Affiliations:** 1 Life Science & Health, Centrum Wiskunde & Informatica, Amsterdam, Netherlands; 2 Genome Data Science, Faculty of Technology, Bielefeld University, Bielefeld, Germany

**Keywords:** Genome assembly, Haplotype, Virus, SARS-CoV-2, Long reads

## Abstract

Haplotype-resolved de novo assembly of highly diverse virus genomes is critical in prevention, control and treatment of viral diseases. Current methods either can handle only relatively accurate short read data, or collapse haplotype-specific variations into consensus sequence. Here, we present Strainline, a novel approach to assemble viral haplotypes from noisy long reads without a reference genome. Strainline is the first approach to provide strain-resolved, full-length de novo assemblies of viral quasispecies from noisy third-generation sequencing data. Benchmarking on simulated and real datasets of varying complexity and diversity confirm this novelty and demonstrate the superiority of Strainline.

**Supplementary Information:**

The online version contains supplementary material available at (10.1186/s13059-021-02587-6).

## Background

Viruses such as HIV, ZIKV, and Ebola lack proofreading mechanisms when they replicate themselves with RNA-dependent RNA polymerase (RdRp) [1, 2]. Therefore, they are characterized by high mutation rates, and commonly populate hosts as a collection of closely related strains which differ by only small amounts of variants, and which together are referred to as viral quasispecies [3]. The genetic diversity of viral quasispecies plays an important role in viral evolution. Among others, it contributes to tissue tropism, virus transmission, disease progression, virulence and drug/vaccine resistance [1, 4–6]. In addition, biological functionalities or phenotypic appearance can differ substantially across different strains [7]. Currently, the COVID-19 pandemic puts the necessity to monitor the outbreak of viruses, to track their evolutionary history, and to develop effective vaccines and drugs in the spotlight of greater public interest. To accurately account for these issues, accurate reconstruction of strain-resolved genomes can be very helpful, if not even necessary.

It is the general, ultimate goal of viral quasispecies assembly to reconstruct the individual, strain-specific haplotypes at their *full length*. Further, along with strain identity-preserving sequence, accurate *estimates of strain abundances* are required for full quantification of infections at the RNA/DNA level. Notwithstanding the short size of virus genomes, it is still a challenge because within a viral quasispecies (i) closely related strains share plenty of near-identical genomic fragments, (ii) single strains are affected by repetitive regions [8], (iii) the number of strains is unknown, and (iv) the abundances of strains vary across the strains, which is further aggravated by read coverage fluctuations along the genomes.

So far, existing methods for viral quasispecies assembly can be classified into *reference-based* approaches on the one hand and de novo (reference free) approaches on the other hand; see [9] for a recent review of related approaches. Reference-based methods such as ShoRAH [10], PredictHaplo [11] and CliqueSNV [12] require high quality reference for reliable reconstruction of strains and, apart from rare exceptions [11, 12], mainly have been specializing in processing relatively error-free short read data. Importantly, high quality reference genomes may not be available precisely when they are needed the most: very often, new outbreaks of known viruses are caused by virus variants that significantly deviate from curated reference sequence [13, 14]. Last but not least, reference-guided methods are prone to introducing biases and can be blind with respect to crucial variant-related details in genomic regions of particular interest [15, 16].

De novo (reference free) viral quasispecies assembly tools, such as SAVAGE [16] or viaDBG [17], both are able to employ overlap and de Bruijn graph-based techniques to assemble NGS reads into haplotype-specific contigs (a.k.a. haplotigs), where the two assembly paradigms, overlap vs de Bruijn graph based, come with different advantages and disadvantages. The resulting contigs of these short read based approaches tend to be too short to span genomes at their full length. The reason are sequence patches that are shared by different strains (and also repetitive areas within strains), which induce ambiguities that cannot be overcome by short reads themselves. For computing full-length genomes, one can try to leverage the strain-specific abundances, which allows to bridge contigs across otherwise ambiguous stretches of sequences. To this end, methods such as Virus-VG [18] and VG-Flow [19] have been developed, the latter approach of which introduced flow variation graphs as a computational concept of potential greater value. Because the runtime is polynomial in the length of the genomes, VG-Flow [19] can also be used for bacteria sized genomes.

We recall that all these existing approaches focus on viral haplotype reconstruction from *short and accurate next-generation sequencing (NGS) reads*, as generated most prominently by Illumina platforms. Again, the fact that short reads fail to span inter- and intra-genomic identical regions crucially hampers the process of reconstructing full-length viral haplotypes. Leveraging strain-specific abundances, as implemented by VG-Flow [18] for example, are not necessarily able to output full-length, strain-specific assembled sequence for certain viruses, such as ZIKV and Polio.

*Quite apparently, virus genome assembly methods have approached their limits when operating with short read NGS data.* Processing long and noisy third-generation sequencing (TGS) data, such as generated by Pacific BioSciences (PacBio), performing single-molecule real-time (SMRT) sequencing, and Oxford Nanopore Technologies (ONT), performing nanopore sequencing, as the currently two most popular sequencing platforms, offers rescue.

The length of TGS reads ranges from several Kbp to hundreds of Kbp, or even to ∼Mbp [20]. TGS reads enable to span intra-genomic repeats and areas shared by different genomes, hence cover regions that are unique to single strains [8]. So, in comparison with NGS reads, TGS reads have considerably greater potential to resolve ambiguities across different strains. The drawback of TGS reads are the elevated error rates they are affected with. Unlike for NGS platforms (sequencing error rate <1*%*), error rates of PacBio CLR and ONT reads range from 5 to 15%, which raises the issue of sequencing errors to a greater order of magnitude.

There are a handful of de novo assembly methods that specialize in processing error-prone long reads such as FALCON [21], Canu [22], Flye [23], Wtdbg2 [24], and Shasta [25], all of which have been published fairly recently. None of these approaches makes a decided attempt to generate haplotype-(strain-) resolved genomic sequence. Rather, these approaches choose to output *consensus* sequence, as a summary across several or all haplotypes/strains in the mix. In other words, all of the de novo assemblers presented in the literature so far fall under the category “generic (or consensus) assembler”.

In addition, metaFlye, originally designed to perform assembly of metagenomes, operates at the level of species [26], so neglects to resolve individual genomes at the level of strains.

*In conclusion, haplotype-aware assembly of viral quasispecies from erroneous long reads can still be considered an unsolved problem: no method is able to address the issue satisfyingly.*

Here, we pursue a novel strategy to resolve the issue. In that, to the best of our knowledge, our approach is the first one to accurately reconstruct the haplotypes of viral quasispecies from third-generation sequencing reads. We recall that processing long TGS reads appears to be the only current option to reconstruct genomes at the level of strains, for the majority of the currently predominant viruses.

In a brief description (see below for details), our novel strategy consists of *local de Bruijn graph-based assembly* in a first step that addresses to wipe out errors. Subsequently, we turn our attention to an overlap graph-based scheme by which to iteratively extend haplotype-specific contigs (haplotigs) into full-length haplotypes. After a filtering step that removes artifacts and preserves true sequence, our approach outputs a set of haplotypes—a large fraction of which appear to have reached full length—together with the relative abundances of the haplotypes within the mix of haplotypes.

We evaluate our approach on various virus datasets that have been approved earlier in the literature. For each dataset, we process both PacBio CLR reads and ONT reads, as the two most predominant types of TGS reads. Benchmarking results on both simulated and real data confirm our claims: our approach accurately reconstructs all full-length haplotypes and delivers sufficiently accurate estimates of their relative abundances.

We also compare our approach with the current state of the art. We recall however that none of the current approaches decidedly addresses the issue of strain-resolved viral quasispecies assembly from long reads. As a consequence, our approach outperforms the state of the art rather drastically. Our approach has its greatest advantages in terms of haplotype coverage, reaching nearly 100% on the majority of datasets. Other methods never get beyond 60–85% (if they get there at all; in particular on ONT data, alternative approaches reach their limit substantially earlier). Further marked advantages are assembly contiguity (measured as per N50 or NGA50) and accuracy (expressed by low error rates and little misassembled contigs). A currently particularly interesting application scenario is the assembly of haplotype-resolved genomes of SARS-CoV-2, because strain resolution will sharpen our understanding about mutation rates and evolutionary development of the virus. Also in this scenario of particular current interest, our approach demonstrates to outperform all existing approaches by fairly large margins.

## Results

We have designed and implemented Strainline, a novel approach that implements the strategy as sketched above. We will describe Strainline in full detail in the following.

In a short summary, Strainline reconstructs full-length, strain-resolved viral haplotypes from noisy long read (TGS read) sequencing data. Strainline is a de novo assembler, so does not have to rely on available reference sequence. Therefore, Strainline operates free of biases induced by prior knowledge, which has been pointed out in earlier work as a notorious source of issues.

In this section, we provide a high-level description of the workflow and evaluate its performance on both simulated and real data, in comparison with existing state-of-the-art tools. In our comparisons, we focus on both generic and metagenome assembly approaches that are able to process long reads without having to rely on reference sequence, which matches the conditions under which Strainline is able to operate.

### Approach

**Fig. 1:**
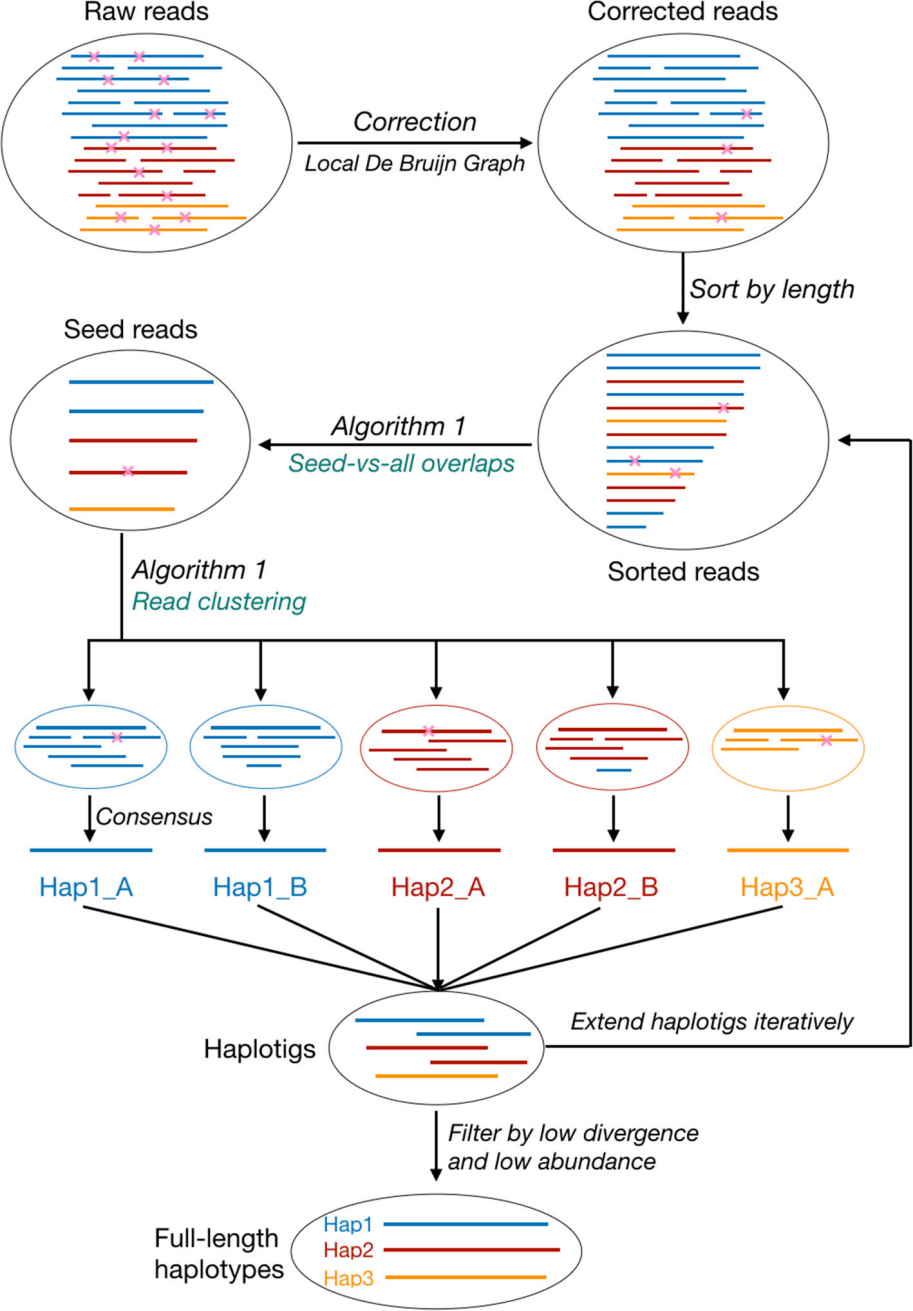
The workflow of Strainline. The reads with different colors are from different haplotypes or strains. The pink fork represents the sequencing error, i.e. mismatch, insertion or deletion. The two steps *Seed-vs-all overlaps* and *Read clustering* are executed simultaneously (see Algorithm 1 for details). Hap1_A and Hap1_B denote two subsequences (not full-length) of haplotype 1 (Hap1), the same for Hap2_A and Hap2_B. There still may be very few remaining sequencing errors in corrected reads such as in the corresponding read cluster of Hap1_A, and incorrectly clustered reads such as the ’blue read’ in the corresponding read cluster of Hap2_B. Nevertheless, these errors will be eliminated through *Consensus* step

See Fig. 1 for an illustration of the overall workflow of Strainline. Here, we describe the workflow briefly. For detailed descriptions of the individual steps, we refer to the “Methods” section.

Strainline consists of three stages. The first stage addresses to correct sequencing errors in the raw long reads, for which it employs *local de Bruijn graph assembly*. The second stage addresses to iteratively extend haplotype-specific contigs into full-length haplotype-specific genomes, based on an overlap based strategy. The third stage, finally, is for filtering the resulting contigs so as to remove haplotypes of too low divergence in comparison with others (so likely reflect errors instead of strain-specific variation), or too low abundance (so likely reflect artifacts). The eventual output is a set of full-length haplotypes along with their corresponding relative frequencies, clear of errors and artifacts.

In general, de Bruijn graph-based approaches tend to be inappropriate for TGS read data, because of the elevated error rates that apply. Rather unexpectedly, we found a (local) de Bruijn graph-based approach, originally developed for long genomes, to effectively work for genomes of tens of thousands of nucleotides in length when provided with sufficiently deep coverage [27]. Apparently, the superiority of the approach when dealing with virus genome settings had passed unnoticed earlier.

**Fig. 2:**
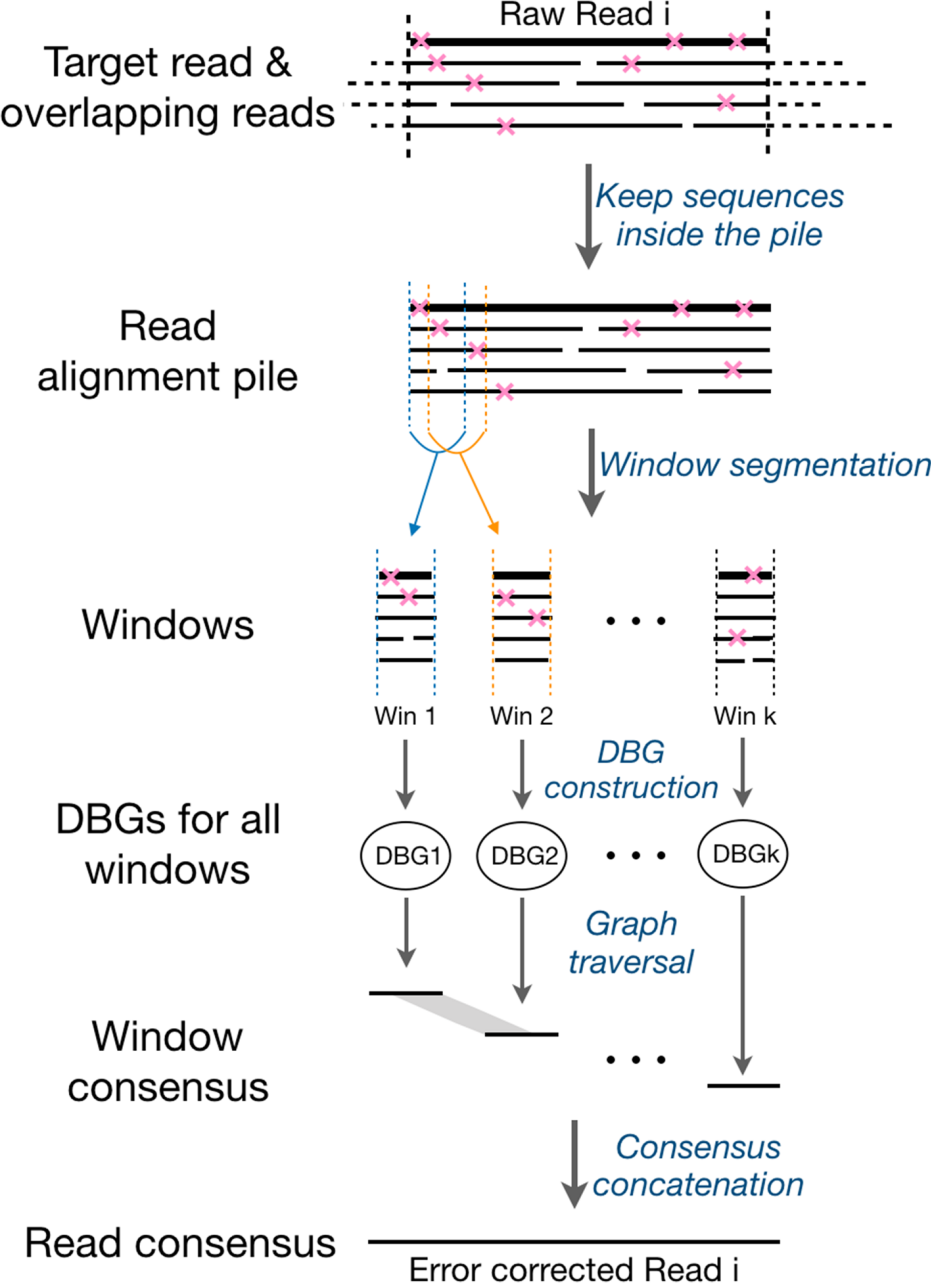
The schematic diagram for the sequencing error correction procedure of raw reads. In the top region, the bold solid line denotes the target raw read *i*, and the overlapping reads of the target read *i* are drawn dashed outside of the read alignment pile and solid inside of it. The pink fork represents the sequencing error, i.e. mismatch, insertion or deletion. The read alignment pile is split into *k* small windows, representing as Win 1, Win 2,... Win *k*. DBG is short for de Bruijn Graph. Window consensus refers to the highest scoring sequences (see main text for explanations) through the DBGs of small windows over the read alignment pile. The region filled with gray rhombus between two window consensus denotes the overlap between them (30bp). We perform the error correction step for each raw read

See Fig. 2 for the following. Given a target read to be corrected, the corresponding strategy considers the reads that overlap the target read, where overlaps are determined based on evaluating canonical k-mers (“Target read & overlapping reads” in Fig. 2). The resulting overlapping reads together with its target read form a read alignment pile that is divided into small windows (“Read alignment pile” and “Windows” in Fig. 2). Subsequently, a de Bruijn graph is constructed for each such small window (“DBGs for all windows” in Fig. 2)). Based on evaluating this de Bruijn graph, an optimal consensus sequence is determined, which reflects the error corrected, true sequence of the target read (see “Window consensus” and “Read consensus” in Fig. 2).

The second stage falls into two sub-steps. Firstly, Strainline determines read clusters where each of the clusters reflects a collection of reads that overlap each other in terms of genomic position (computed by “Algorithm 1” in Fig. 1). Secondly, Strainline uses a partial order alignment (POA) algorithm to yield a consensus sequence for each read cluster. These consensus sequences are expected to be haplotype-specific contigs (haplotigs). The haplotigs are then iteratively extended into full-length haplotypes.

The third and final stage is to filter haplotypes having very low divergence or very low relative abundance. Most likely, such haplotypes were introduced due to redundant or spurious sequences. See the “Methods” section for full details on each of the stages involved in the overall workflow.

### Datasets

For the following, see also Table 1 for characteristics of both simulated and experimental data, and see Additional file 1: Table S1 in the Supplement for accession numbers of the corresponding source genomes.

**Table 1:** Characteristics of benchmarking data sets

Virus mixture	Virus type	#Strain	Genome size (bp)	Coverage	Divergence (%)	Strain abundance (%)
*Simulated*						
5-strain HIV	HIV-1	5	9478–9719	20,000 ×	2.7–5.6	10, 15, 20, 25, 30
6-strain Poliovirus	Poliovirus-2	6	7428–7460	20,000 ×	0.2–5.5	2, 4, 8, 16, 20, 50
6-strain Poliovirus (la1)	Poliovirus-2	6	7428–7460	20,000 ×	0.2–5.5	0.1, 1, 2, 8, 20, 68.9
6-strain Poliovirus (la2)	Poliovirus-2	6	7428–7460	20,000 ×	0.2–5.5	0.01, 0.1, 1, 2, 8, 88.89
10-strain HCV	HCV-1a	10	9273–9311	20,000 ×	2.8–7.4	5, 6, 7, 8, 9, 11, 12, 13, 14, 15
15-strain ZIKV	ZIKV	15	10,251–10,269	20,000 ×	1.1–15.1	2, 4, 5, 5, 5, 6, 6, 6, 7, 7, 8, 8, 9, 10, 12
5-strain SARS-CoV-2	SARS-CoV-2	5	26,574–29,903	20,000 ×	0.3–1.1	10, 15, 20, 25, 30
5-strain SARS-CoV-2 (la)	SARS-CoV-2	5	26,574–29,903	20,000 ×	0.3–1.1	0.1, 1, 5, 10, 83.9
*Experimental*						
5-strain PVY (Mock)	PVY	5	9694–9701	5800 ×	3.6–21.6	9.3, 12.7, 21.1, 24.4, 32.5
SARS-CoV-2 (Real)	SARS-CoV-2	-	-	12,000 ×	-	-

For each benchmarking data set, we specify the name of virus mixture, virus type, number of strains in the mixture, range of genome size, total sequencing coverage, pairwise divergence, and strain abundance spectrum. The pairwise divergence is equal to 1−ANI, where ANI (Average Nucleotide Identity) is calculated by FastANI [31]. In experimental data sets, 5-strain PVY is a mock community, that is the sequencing data is real, but the mixture is synthetic, whereas SARS-CoV-2 (Real) is a real sample so there is no ground truth for the strains. The data sets 6-strain Poliovirus (la1) and 6-strain Poliovirus (la2) are similar with 6-strain Poliovirus, except the lowest abundance (la) of strains extends to 0.1% and 0.01%, respectively. The data set 5-strain SARS-CoV-2 (la) is similar with 5-strain SARS-CoV-2 except the lowest abundance (la) of strains extends to 0.1%

#### Simulated data

We simulated various datasets using both PacBio and ONT long read sequencing technologies, yielding the common (PacBio CLR and ONT), high sequencing error rates (5% ∼15%), using PBSIM V1.0.3 [28] and NanoSim V2.6.0 [29], as approved read simulators. We used four virus mixture datasets (HIV, Poliovirus, HCV, and ZIKV), similar in terms of composition of strains to those presented by [18]. We further generated one additional dataset reflecting a SARS-CoV-2 quasispecies, composed of 5 strains (note that the number of SARS-CoV-2 strains that affect one individual is entirely unclear at this point, because of the lack of analysis tools). Notably, it is very common to perform ultra-deep sequencing supported by the short length of the viral genome (7 ∼30kbp in our cases) [16, 18, 30]. Therefore, we uniformly set the overall sequencing depth (the sum of average depth of each strain) as ∼20,000× on all simulated datasets, which reflects common real world practice.

Additionally, in order to evaluate the effect of sequencing coverage, we generated 5-strain HIV datasets at an error rate of 10% and varying coverage, of overall depths of 500×,1000×,2000×,5000×,10,000× and 20,000×, respectively, while relative frequencies of strains did not change. More details about simulated data are shown in Data simulation.

**5-strain HIV mixture.** This dataset consists of five known HIV-1 strains (YU2, NL43, JRCSF, HXB2, 896), as originally presented in [30]. Strains were simulated at relative abundances between 10 and 30%, i.e. a sequencing coverage of 2000× to 6000× per strain. This virus mixture is one of the most challenging datasets, because the highly repetitive regions in the HIV genome, which usually hamper the performance of short-read-based assemblers [16, 18, 30].

**6-strain Poliovirus mixture.** This mixture contains six strains of Poliovirus (Type 2), with exponentially increasing relative abundances from 2% to 50%. The haplotype sequences were downloaded from the NCBI database. In addition, we simulated *la1* and *la2*, as two more data sets reflecting 6 Poliovirus strains, where la1 contains strains of abundance as low as 0.1% and la2 contains strains of abundance as low as 0.01%; see Table 1 for a full description of these data sets in terms of strains and their abundances.

**10-strain HCV mixture.** This mixture contains ten strains of hepatitis C virus (HCV), Subtype 1a, with relative frequencies varying from 5 to 15% per haplotype. The haplotype sequences were also obtained from the NCBI database.

**15-strain ZIKV mixture.** This mixture consists of fifteen strains of Zika virus (ZIKV), of which three master strains were obtained from the NCBI database and four mutants were generated per master strain by randomly introducing mutations. The relative frequency of strains varies between 2 and 12%.

**5-strain SARS-CoV-2 mixture.** This mixture consists of five strains of SARS-CoV-2, with the relative frequencies varying from 10% to 30%. The true haplotype sequences (high quality without N bases) were extracted from different regions (namely, Belgium, Egypt, Oman, USA, China) in the GISAID (https://www.gisaid.org/) database. In addition, resembling the Poliovirus data sets, we simulated *la*, as a data set containing 5-strains SARS-CoV-2 where two strains come at abundances as low as 1% and 0.1%, respectively; see again Table 1 for a detailed description.

#### Experimental data

To evaluate our method on real sequencing data, we downloaded two experimental datasets for benchmarking analysis.

**5-strain PVY mixture.** This dataset consists of five Potato virus Y (PVY) strains. The true sequences of five strains were accessed from GenBank under accession numbers MT264731–MT264741. The corresponding real ONT reads were obtained from the SRA database under BioProject PRJNA612026, as recently presented in [32]. We downloaded long read sequencing data for each strain and then mixed them together to generate a pseudo virus mixture (mock community), where strains have relative frequencies varying from 9 to 33% and the total sequencing depth is approximate 5800 ×.

**SARS-CoV-2 real sample.** This dataset is Oxford Nanopore sequencing (GridION) data of a real SARS-CoV-2 sample, as downloaded from the SRA database: SRP250446. The N50 of the length of the reads is 2.5 kbp, the average sequencing error rate is approximately 10% and the average sequencing coverage is about 12,000 ×.

### Benchmarking: alternative approaches

We recall that Strainline is unique insofar as it is the first approach to determine the haplotype/strain-specific genomes of viruses from long reads de novo. For the sake of a meaningful comparison, we chose long read de novo assemblers that are designed to deal with mixed samples (in other words, designed for metagenome assembly), such as Canu [22] and metaFlye [26], on the one hand, and generic (consensus) de novo assemblers, such as Wtdbg2 [24] and Shasta [25] on the other hand. Of those, we subsequently excluded metaFlye, because it failed to perform the assemblies on our datasets^1^. Shasta returned too many fragmented contigs, indicating that no real assembly was computed. For fairness reasons—we recall that all tools were originally designed for different purposes, but not strain aware virus genome assembly—we excluded metaFlye and Shasta from further consideration. For Canu, we used the parameters recommended for metagenome assembly, and we ran Wtdbg2 with default parameters. The output contigs were then subject to being evaluated.

In addition, we also benchmarked reference-guided methods such as PredictHaplo [11] and CliqueSNV [12], which can reconstruct haplotypes from long-read sequencing data. However, we failed to run PredictHaplo on our long-read data sets (we have reported the so far unresolved issue at https://github.com/cbg-ethz/PredictHaplo/issues/1). For CliqueSNV, we reported results for PacBio data since CliqueSNV has not been validated on ONT data by far as much as PacBio (and Illumina) data.

### Performance evaluation

#### Assembly metrics

In the evaluation, we considered all relevant categories, as output by QUAST V5.1.0 [33], as a prominent assembly evaluation tool. As is common, we discarded contigs of length less than 500 bp from the output of all tools. In particular, we ran the metaquast.py program with the option –unique-mapping appropriately taking into account that our data sets reflect mixed samples. In the following, we briefly define the metrics we are considering.

**Haplotype coverage (HC).** Haplotype coverage is the percentage of aligned bases in the ground truth haplotypes covered by haplotigs. Haplotype coverage is commonly used to measure the completeness in terms of genome diversity of the assembly.

**N50 and NGA50.** We also consider N50 and NGA50 to measure assembly contiguity, as per their standard definitions: N50 is the maximum value such that all contigs of at least that length cover at least half of the assembly, and NGA50 is the maximum value such that the contigs of at least that length cover at least half of the reference sequence when aligned against it (after breaking contigs at misassembly events and trimming all unaligned nucleotides); here, the reference sequence is taken to reflect the concatenation of all strain-specific genomes from which reads were simulated, or the canonical choices of reference sequences for real data otherwise.

**Error rate (ER).** The error rate is the fraction of mismatches, indels and N’s (i.e. ambiguous bases) in the alignment of the contigs with the reference sequences.

**Misassembled contigs proportion (MC).** If a contig involves at least one misassembly event, it is counted as *misassembled contig*. A misassembly event is given when contigs align with a gap or overlap of more than 1kbp, align to different strands, or even different haplotypes. As MC, we report the percentage of misassembled contigs relative to the overall number of output contigs.

**Precision and recall.** Because it was found helpful in evaluating virus genome assemblies earlier, we also report precision and recall. While precision refers to the fraction of contigs that align to the correct strain-specific sequence, recall refers to the fraction of strains that have a correctly aligned contig. Therefore, the edit distance of the alignment of the contig with the reference sequence must not exceed a threshold *d*, which can vary (e.g., 0%, 1%, 2%, 3%, 4%, 5%, see Additional file 1: Figures S1–S3). In Results in the main text, the most stringent threshold 1% is used).

#### Haplotype abundance evaluation

Furthermore, we use two further metrics equally suggested in prior work as helpful for evaluating virus genome assembly quality [18, 19]. Namely, we report the absolute frequency error (AFE) and the relative frequency error (RFE), which measure the deviation of the estimated abundances from the true abundances of the haplotypes. Let *k* be the number of true haplotypes. For a haplotype *i*∈{1,...,*k*}, let $\widehat {a_{i}}$ and *a*_*i*_ be the estimated and true abundance of haplotype *i*, respectively. To determine $\widehat {a_{i}}$, we first collect all haplotigs that get aligned with *i*, and then add up the abundances of these haplotigs. Let further $I=\left \{ i\in [k]:\widehat {a_{i}}>0 \right \}$ be all haplotypes that by their abundance were estimated to exist. One then calculates: $\text {AFE} = \frac {1}{|I|}\sum _{i\in I}^{}|a_{i}-\widehat {a_{i}}|, \text {RFE} = \frac {1}{|I|}\sum _{i\in I}^{}|a_{i}-\widehat {a_{i}}|/a_{i}$.

### Benchmarking results

We performed benchmarking experiments including all methods on the simulated and experimental data as described above, for both PacBio CLR and Oxford Nanopore reads. In a short summary of the results *ex ante*, Strainline manages to reconstruct all full-length haplotypes accurately from most of the mixed viral samples. Because truly specializing competing approaches are lacking, Strainline performs best in comparison with any alternative approach, by quite drastic margins.

**Simulated PacBio CLR data.** See Table 2 and Additional file 1: Table S3. Strainline yields near-perfect assemblies on six out of eight datasets (HIV, Poliovirus, Poliovirus (la1), HCV, ZIKV, and SARS-CoV-2). That is, all ground truth haplotypes in the mixed samples are reconstructed to their full extent (HC ≈100%, recall =100%), at very low error rates (0.002% ∼0.3%) and without misassemblies. It also obtains the exact number of true haplotypes on four datasets (precision is 100%), and overestimates the number of haplotypes in the 6-strain Poliovirus (la1) mixture (precision =75%, recall =100*%*) and 5-strain SARS-CoV-2 mixture (precision =71.4%, recall =100*%*). Strainline fails to reconstruct all haplotypes on two datasets, namely, 6-strain Poliovirus (la2) (HC =65.3*%*) and 5-strain SARS-CoV-2 (la) (HC =60.8*%*). We recall that these two datasets are particularly challenging because they contain strains of very low abundance (0.01%, 0.1%) and divergence (0.2%, 0.3%); see Table 1 for details. Of note, while Strainline still accurately reconstructs Poliovirus strains of 0.1% (amounting to 20 × coverage), Strainline struggles to do that for SARS-CoV-2 strains. The explanation for this effect is the fact that the SARS-CoV-2 genome is 4 times longer than the Poliovirus genome (∼30,000 bp versus ∼7500 bp). The expected amount of coverage breaks for a 20 × covered strain in a 30,000 bp genome is too large to still allow for accurate full-length reconstruction, while still being feasible for genomes of 7500 bp in length. In addition, the low divergence increases the probability of ambiguous stretches in longer genomes. In comparison, both de novo assemblers (Canu and Wtdbg2) and reference-guided methods (CliqueSNV) struggle to reconstruct the haplotypes on all eight datasets, with Canu achieving only 49% to 85.9% haplotype coverage on these datasets. Canu further achieves 100% recall on SARS-CoV-2, but only 50% to 80% recall on the other seven datasets (see Additional file 1: Figure S1). Notably, Strainline outperforms other tools in terms of precision on five datasets except that Canu achieves better precision on the 5-strain SARS-CoV-2 dataset and Wtdbg2 achieves better precision on 6-strain Poliovirus (la1), 5-strain SARS-CoV-2 and SARS-CoV-2 (la) datasets. The reason for Canu’s successes on the 5-strain SARS-CoV-2 dataset in terms of precision and recall is the fact that its assembly is heavily fragmented (Canu generates 16 fragmented contigs, at an N50 of 12419). This puts Canu’s achievements into a different context (and indicates that precision and recall have to be taken with a certain amount of care in the evaluation of assembly performance). Wtdbg2 basically generates a single consensus genome sequence rather than keeping haplotype information. Consequently, it merely obtains 10.7% to 20.6% haplotype coverage at high error rates, for example, 1.8%, 5.1%, and 1.7% on HIV, HCV, and ZIKV datasets, respectively. This also puts the fact that Wtdbg2 achieves great precision into context: while great precision is supported by computing only a single haplotype, recall suffers decisively. CliqueSNV also only reconstructs a fraction of the haplotypes making part of the virus mixtures (HC: 10∼77*%*), regardless of the choice of reference genome used: neither the most abundant strain nor any reasonable bootstrap consensus genome, as generated by Wtdbg2 or Strainline, for example, enhance CliqueSNV’s performance significantly. The explanation for CliqueSNV’s performance rates are the large amount of haplotype-specific SNP’s, which induces bottlenecks during the computation of cliques, as inherent to the algorithm of CliqueSNV (we thank the authors for the personal communication).

**Table 2:** Benchmarking results for simulated PacBio CLR reads. *HC* haplotype coverage, *ER* error rate (mismatches + indels + N’s), *MC* misassembled contigs proportion. NGA50 is labeled with “-” if the uniquely aligned blocks cover less than half of the reference length. The total sequencing coverage in this table is 20,000 ×. Note that CliqueSNV is a reference-based method, whereas the others are de novo assemblers. For running CliqueSNV, we have tried various strategies (see Additional file 1: Table S2) but only the results of the best strategy are reported here. * If contigs are full-length, this number represents the estimated number of haplotypes or strains in the virus mixture. ^*†*^ Wtdbg2 consensus as reference, using reads corrected by Strainline error correction. ^*‡*^ High quality reference (the highest abundant strain) using reads corrected by Strainline error correction

	#Contigs*	HC (%)	N50 (bp)	NGA50 (bp)	ER (%)	MC(%)
*5-strain HIV mixture*						
Strainline	5	99.9	9697	9697	0.002	0.0
Canu	5	84.5	8227	8170	0.409	20.0
Wtdbg2	1	15.5	7419	-	1.820	0.0
CliqueSNV ^*†*^(reference based)	8	77.2	7419	7419	1.106	0.0
*6-strain Poliovirus mixture*						
Strainline	6	99.9	7449	7444	0.074	0.0
Canu	6	62.7	7040	6399	0.538	0.0
Wtdbg2	1	14.7	6575	-	0.244	0.0
CliqueSNV ^*‡*^(reference based)	4	49.9	7452	7438	0.433	0.0
*10-strain HCV mixture*						
Strainline	10	99.9	9294	9292	0.056	0.0
Canu	11	76.9	7703	7174	0.351	0.0
Wtdbg2	2	13.6	7698	-	5.077	0.0
CliqueSNV ^*‡*^(reference based)	1	10.0	9310	-	1.963	0.0
*15-strain ZIKV mixture*						
Strainline	15	99.6	10,238	10,238	0.021	0.0
Canu	13	55.7	10,233	7129	0.189	0.0
Wtdbg2	2	10.7	8773	-	1.693	0.0
CliqueSNV ^*‡*^(reference based)	1	6.7	10,268	-	1.627	0.0
*5-strain SARS-CoV-2 mixture*						
Strainline	7	98.6	29,017	29,009	0.047	0.0
Canu	16	85.9	12,419	25,137	0.078	0.0
Wtdbg2	1	20.6	29,158	-	0.360	0.0
CliqueSNV ^*‡*^(reference based)	1	21.1	29,903	-	0.007	0.0

Considering results for the 3 mixtures containing strains of extremely low abundance, la1 and la2 for Polio and la for SARS-CoV-2, see Additional file 1: Table S3, we find that Strainline still reconstructs all strains in la1 (HC: 98%, Recall=100%, ER=0.3%), which points out that Strainline is able to reconstruct strains of 20 × coverage (abundance 0.1% times 20,000 × coverage overall), albeit at the expense of overestimating the number of haplotypes (precision = 75%). Strainline eventually struggles to reconstruct all strains in data sets la2 (Polio; HC=65.3%) and la (SARS-CoV-2; HC=60.8%). This translates into Strainline failing to reconstruct strains of coverage 2 × (0.01% times 20,000 ×). Of course, 2 × reflects a coverage rate that induces various coverage breaks along the genome, which prevents to assemble the corresponding sequence already in theory. The low divergence of strains (0.2%, 0.3%) in these data sets adds to the difficulties induced by the very low coverage.

All other methods struggle already on la1, on which Strainline still exhibited good performance rates. Canu, as the second best performing tool, for example, achieved haplotype coverage of 59% and 49% on la1 and la2, respectively.

**Simulated ONT data.** Table 3 displays the benchmarking results for Oxford Nanopore reads assembly. Again, Strainline yields near-perfect results on all five datasets. All true haplotypes are reconstructed (HC ≈100%, recall =100%) with extremely low error rate (0.023 ∼0.081%), and without misassemblies. Moreover, it achieves the exact number of true strains on three datasets (HIV, Poliovirus and HCV, precision =100%), and overestimates the number of haplotypes by one on the 15-strain ZIKV and the 5-strain SARS-CoV-2 mixtures (precision is 100% and 83.3%, respectively).

**Table 3:** Benchmarking results for simulated Oxford Nanopore reads. *HC* haplotype coverage, *ER* error rate (mismatches + indels + N’s), *MC* misassembled contigs proportion. NGA50 is labeled with ’-’ if the uniquely aligned blocks cover less than half of the reference length. The total sequencing coverage in this table is 20,000 ×

	#Contigs	HC (%)	N50 (bp)	NGA50 (bp)	ER (%)	MC(%)
*5-strain HIV mixture*						
Strainline	5	99.9	9702	9702	0.081	0.0
Canu	2	35.8	18,151	7634	1.730	50.0
Wtdbg2	1	18.9	9046	-	1.327	0.0
*6-strain Poliovirus mixture*						
Strainline	6	100.0	7454	7453	0.051	0.0
Canu	1	16.6	7446	-	0.646	0.0
Wtdbg2	-	-	-	-	-	-
*10-strain HCV mixture*						
Strainline	10	99.9	9294	9294	0.023	0.0
Canu	1	10.0	9279	-	2.619	0.0
Wtdbg2	2	18.4	8567	-	1.336	0.0
*15-strain ZIKV mixture*						
Strainline	16	98.3	10,244	10244	0.026	0.0
Canu	1	6.6	10,251	-	0.459	0.0
Wtdbg2	3	17.1	9490	-	0.564	0.0
*5-strain SARS-CoV-2 mixture*						
Strainline	6	99.9	29,299	29071	0.041	0.0
Canu	10	66.3	18,003	9492	0.062	0.0
Wtdbg2	1	19.0	26,767	-	0.586	0.0

In comparison, Canu obtains 16.6%, 10.0%, and 6.6% haplotype coverage on Poliovirus, HCV, ZIKV datasets, respectively, which indicates that Canu does not operate in a strain-specific manner. On HIV and SARS-CoV-2, Canu achieves better haplotype coverage, while still missing a considerable proportion of strains (HC = 35.8% and 66.3%, respectively). While Canu has 100% recall on SARS-CoV-2, only 0% to 20% recall are achieved on the other four datasets (see Additional file 1: Figure S2). Canu also shows relatively high error rates in the HIV and HCV assemblies (1.7% and 2.6%; 21 and 114 times higher than Strainline, respectively). For Poliovirus and ZIKV datasets, Canu displays about 15 times higher error rates in comparison with Strainline.

Wtdbg2 only yields 17% to 19% haplotype coverage on four datasets (except Poliovirus) at relatively high error rates (e.g., 1.3% on HIV and HCV). Wtdbg2 failed to run on the Poliovirus dataset, so no results are shown.

**Experimental data.** See Table 4 for results on the 5-strain PVY mixture. Also here, Strainline reconstructs the great majority of strain-specific sequences (HC=97.9%, recall=60%). Importantly, recall is 100% if edit distance is set to 3%. Similarly, Strainline overestimates the number of haplotypes by two, but achieves perfect precision (100%) when operating at edit distance 3%.

**Table 4:** Benchmarking results for real Oxford Nanopore reads. *HC* haplotype coverage, *ER* error rate (mismatches + indels + N’s), *MC* misassembled contigs proportion. NGA50 is labeled with ’-’ if the uniquely aligned blocks cover less than half of the reference length. Note that the metrics for the SARS-CoV-2 real sample in this table are not necessarily correct but for reference only, because the ground truth is unknown and we only used the sequence of Wuhan-Hu-1 (NC_045512) as the truth for comparison. ^*†*^ Strainline consensus as reference, using reads corrected by Strainline error correction. * Sometimes NGA50 still reports a value (5456 bp) even if HC <50*%* because contigs have overlaps (See https://github.com/ablab/quast/discussions/ 174 for the detailed explanation)

	#Contigs	HC (%)	N50 (bp)	NGA50 (bp)	ER (%)	MC(%)
*5-strain PVY mixture*						
Strainline	7	97.9	9538	9548	0.956	0.0
Canu	3	39.9	9665	5456*	0.105	0.0
Wtdbg2	2	26.0	7632	-	4.931	0.0
*SARS-CoV-2 (SRP250446)*						
Strainline	1	99.9	29,565	29,565	0.832	0.0
Canu	-	-	-	-	-	-
Wtdbg2	1	65.8	19,405	19,396	1.542	0.0
CliqueSNV ^*†*^	1	99.9	29,565	29,565	0.859	0.0

Canu only reconstructs the most abundant two strains at full haplotype coverage and high accuracy (error rate for these two strains is 0.07% and 0.09%). However, Canu misses to cover any of the other three strains (reflected by HC=40% and recall=40%). Strainline, on the other hand, reconstructs the most abundant two strains at their full length and the exact same low error rate as Canu. In addition, unlike Canu, Strainline assembles also the other less abundant three strains at full coverage and sufficiently low error rate (0.18% to 2.7%). Wtdbg2 only generates one single near full-length haplotype and another one short contig (HC=26%) at an error rate of 4.9%.

As for the SARS-CoV-2 real sample (SRP250446), we use the genome sequence of Wuhan-Hu-1 (NC 045512) as the reference for comparison since the ground truth is unknown. Strainline yields one single full-length haplotype (HC=99.9%) at an error rate clearly below the sequencing error rate (0.8%). Wtdbg2 only obtains one fragmented contig (HC=65.8%, N50=19,405) with about two times higher error rate (1.5%). Canu was unable to finish after running for more than ten days on a 48-core computing machine, clearly exceeding acceptable computational resources, so we stopped the job. This explains why no results are shown.

### Haplotype abundance estimation

We also evaluated the accuracy of estimated haplotype abundances on both simulated and real data, see Table 5. Note that Canu and Wtdbg2 do not provide abundance estimation (because of their general setup as consensus assemblers), and CliqueSNV only reconstructs a minority of haplotypes, so no reasonable comparison with other methods can be provided.

**Table 5:** Absolute and relative errors of estimated haplotype abundances by Strainline on different virus mixtures. For each dataset, we present the average error over all assembled strains. Note that for the *SARS-CoV-2 (SRP250446)* real sample, the abundance estimation error is not shown because the ground truth is unknown. *AFE* absolute frequency error, *RFE* relative frequency error

Datasets	AFE (%)	RFE (%)
*Simulated PacBio CLR*		
5-strain HIV	0.04	0.18
6-strain Poliovirus	2.17	48.35
10-strain HCV	0.06	0.80
15-strain ZIKV	0.18	4.93
5-strain SARS-CoV-2	3.40	17.49
*Simulated ONT*		
5-strain HIV	0.27	1.48
6-strain Poliovirus	2.30	51.18
10-strain HCV	0.28	3.05
15-strain ZIKV	0.28	6.10
5-strain SARS-CoV-2	4.14	20.60
*Experimental ONT*		
5-strain PVY	4.48	23.07

Strainline estimates the frequencies for the reconstructed haplotypes at operable accuracy, with absolute frequency error (AFE) of 0.04%/0.27% (PacBio/ONT) on the simulated HIV data, 0.06%/0.28% on the HCV data and 0.18%/0.28% on the ZIKV data, respectively. One observes that the relative frequency errors (RFE) follow a similar pattern. On simulated Poliovirus, SARS-CoV-2 and experimental PVY datasets, Strainline estimates the abundances at moderate accuracy (AFE is between 2.17 and 4.48%). A likely explanation for increased error rates on Poliovirus data is the fact that strain abundances vary exponentially (exponentially increasing from 1.6 to 50.8%). The less accurate estimates on Poliovirus refer mainly to low frequent strains, which naturally causes high relative frequency errors (48%, 51% on PacBio and ONT data). A possible reason for increasing errors of abundance estimates on the simulated SARS-CoV-2 and the experimental PVY datasets is the overestimation of the number of haplotypes in the mixed samples. These findings suggest that accurate haplotype reconstruction goes hand in hand with accurate haplotype abundance estimation. The results are also likely to reflect the current limits in that respect, because coverage fluctuations and partial amounts of reads of shorter length impose certain constraints on estimating haplotype abundance.

### Error correction evaluation

In addition to benchmarking analysis for assemblies, we benchmarked several error correction tools, Daccord [27] (integrated in Strainline) and other widely used tools, such as Racon [34], LoRMA [35], CONSENT [36], and, last but not least, the error correction module of Canu [22]. While Canu and Racon are based on multiple sequence alignments (MSAs), LoRMA and CONSENT are based on combining de Bruijn graphs with MSAs. We experienced that Racon and CONSENT failed to handle the virus data sets. The likely reason is the ultra-high sequencing coverage (∼20,000×), which induces massive amounts of read overlaps, which in turn easily exceeds computational resources in the downstream analysis (programs crashed on 48 cores and 500GB RAM). Benchmarking results for error correction are shown in Additional file 1: Table S4 (PacBio CLR) and Table S5 (ONT). The results show that Daccord achieves 3∼65 times lower error rate on PacBio CLR data, and 2∼5 times lower error rate on ONT data, in comparison with Canu and LoRMA. All three tools keep nearly 100% haplotype coverage (without losing genome information) and show comparable read length (N50). Of note, Daccord’s error correction performance is about an order of magnitude better for PacBio than for ONT data, while still Daccord outperforms other tools also on ONT data. In summary, this justifies to make preferred use of Daccord for correcting errors in long reads used in strain aware virus genome assembly.

Further, we tested the performance of Daccord by varying the size of the local windows, as the boundaries of the de Bruijn graphs to be constructed, from 20 to 200 bp on both PacBio CLR and ONT data, see Additional file 1: Figure S5 for the corresponding results. The experiments show that optimal window sizes range from 30 to 50 bp, where larger window sizes are computationally more demanding. Following these experiments, we determined 40 bp as the most reasonable local window size, kept as the default in Strainline.

### Effects of divergence versus ratio of abundances

**Fig. 3:**
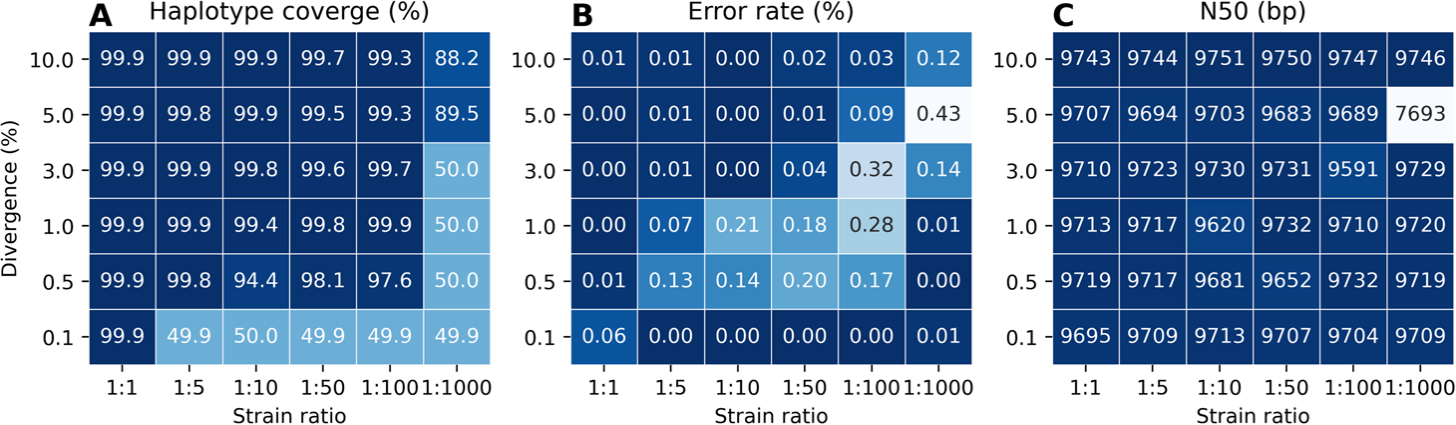
Performance of Strainline with varying strain abundance (= relative frequency) and divergence in two-strain mixtures using simulated PacBio CLR reads. The x, y axis refers to the abundance ratio and the pairwise divergence of two strains in the mixture, respectively. Panels **A**, **B**, and **C** refer to haplotype coverage, error rate (mismatch + indel) and N50 of the resulting assemblies, respectively. The darker colors indicate the better assembly performance

To investigate the effects of divergence and differences in abundance for strains in a virus mixture, we simulated several mixtures consisting of two strains, across all possible combinations of divergence of 0.1%, 0.5%, 1.0%, 3.0%, 5.0%, and 10.0% (where 0.1% is the most and 10.0% is the least challenging) and ratio of abundances of 1:1, 1:5, 1:10, 1:50, 1:100, and 1:1000 (where 1:1 is the least and 1:1000 is the most challenging). In the experiments, we focused on PacBio CLR reads. We then evaluated Strainline in terms of haplotype coverage (HC), error rate (ER), and N50 for each of the possible combinations of divergence and abundance ratio. Results are shown in Fig. 3. As per inspecting N50, one realizes that Strainline reconstructs full-length strains in all cases (apart from divergence 5% and ratio 1:1000, which appears to be an outlier). As for HC, Strainline performs optimally for all combinations of at least 0.5% divergence and abundance ratio 1:100, while requiring a ratio of 1:1 for divergence of 0.1% and divergence at least 5.0% for ratio 1:1000 to reconstruct (the majority) of the two strains. Error rates appear to be suffer for combinations for the extreme cases where still two strains are reconstructed, for example divergence of only 0.5% and strain ratio of 1:100; note that for lower divergence and strain ratio, only one strains gets reconstructed, which however appears to be free of errors. Note as well that also as per ER, the combination of 5.0% divergence and strain ratio of 1:1000 appears to be an artifact.

### Robustness evaluation

To evaluate the robustness of assemblers with respect to random effects induced by the simulation procedure, we repeatedly simulated five *6-strain Poliovirus mixtures*, as a representative data set, for both PacBio CLR and ONT reads. Results are shown in Additional file 1: Figure S4. In summary, Strainline outperforms Canu and Wtdbg2 in terms of robustness with respect to the usual, most relevant assembly metrics, such as haplotype coverage, error rate and N50.

Note that in Fig. S4 (B) for ONT reads Canu achieves lower error rate than Strainline. This is because Strainline generates almost all strains (including the less frequent strains), thus raising the average error rate, whereas Canu can only reconstruct the most abundant strains. In fact, Strainline still achieves lower error rates than Canu when directly comparing it on the most abundant strains.

### Effect of sequencing coverage

To investigate the effect of sequencing coverage on viral quasispecies assembly, we chose a 5-strain HIV mixture, as one of the most challenging datasets suggested in [16, 18]. We simulated PacBio CLR reads with different overall sequencing coverage. Assembly results are shown in Table 6. We observe that Strainline successfully reconstructs all true haplotypes at nearly perfect coverage (HC >99*%*) and great accuracy at sequencing coverage of at least 2000 ×. In that respect, Strainline outperforms all other methods quite substantially. When decreasing sequencing depth below 2000 × (i.e. 1000 to 500 ×), Strainline still achieves haplotype coverage (HC) of approximately 80%, thereby still establishing clear improvements over the alternative approaches. In terms of error rates, Canu catches up with Strainline starting from coverage rates of 2000 × in decreasing order. In terms of all other metrics (such as N50, NGA50 MC), Strainline appears to deliver optimal performance across all coverage rates, which does not apply for the alternative approaches.

**Table 6:** Benchmarking results for 5-strain HIV mixture (PacBio CLR reads) with varying sequencing coverage. *HC* haplotype coverage, *ER* error rate (mismatches + indels + N’s), *MC* misassembled contigs proportion

	#Contigs	HC (%)	N50 (bp)	NGA50 (bp)	ER (%)	MC(%)
*500 ×*						
Strainline	7	78.4	9517	9545	1.603	0.0
Canu	4	32.9	6766	5281	1.447	0.0
Wtdbg2	1	18.0	8687	-	2.295	0.0
*1000 ×*						
Strainline	5	79.2	9557	9554	1.069	0.0
Canu	5	63.6	7207	7139	1.087	0.0
Wtdbg2	1	13.5	6516	-	1.520	0.0
*2000 ×*						
Strainline	6	99.3	9644	9614	0.675	0.0
Canu	5	67.4	8193	7544	0.416	20.0
Wtdbg2	1	16.0	7741	-	1.103	0.0
*5000 ×*						
Strainline	5	99.7	9690	9686	0.311	0.0
Canu	4	69.3	8264	8001	0.537	25.0
Wtdbg2	1	16.5	7950	-	2.115	0.0
*10000 ×*						
Strainline	5	99.8	9696	9691	0.184	0.0
Canu	4	71.7	8844	8524	0.446	25.0
Wtdbg2	1	15.6	7528	-	2.777	0.0
*20000 ×*						
Strainline	5	99.9	9697	9697	0.002	0.0
Canu	5	84.5	8227	8170	0.409	20.0
Wtdbg2	1	15.5	7419	-	1.820	0.0

### Effect of sequencing error rate

To investigate the effect of sequencing error rate on viral quasispecies assembly, we used 5-strain HIV mixtures, as we did for evaluating effects of varying sequencing coverage. We simulated both PacBio CLR and ONT reads with different sequencing error rate (5∼30*%*). Assembly results are shown in Additional file 1: Tables S9 (CLR) and S10 (ONT). We observe that Strainline successfully reconstructs all true haplotypes at nearly perfect coverage (HC ≈100*%*) and correctly estimates the number of haplotypes in all PacBio CLR data sets across varying sequencing error rates (5∼30*%*). On the ONT data sets, Strainline shows similar performance rates for ONT error rates ranging from 5∼25*%*; note that for both PacBio CLR and ONT data, the error rates of Strainline increase on increasing error rates for the reads themselves, albeit only by fairly small amounts, in particular in comparison with the alternative approaches. Only for the ONT dataset of 30% sequencing errors, Strainline experiences losses in terms of performance, the reason of which is likely the somewhat poorer performance of Daccord (as the error correction method used in Strainline) on ONT in comparison with PacBio CLR. Note however that this drawback rather remains a theoretical issue, because ONT is steadily improving in terms of error rates, so 30% rather reflects an artificial scenario in current times.

### Runtime and memory usage evaluation

We performed all benchmarking analyses on a x86_64 GNU/Linux machine with 48 CPUs. The runtime and peak memory usage evaluations for different methods are reported in Additional file 1: Tables S6 and S7. Undoubtedly, Wtdbg2 is the fastest tool, taking only a few seconds and 0.1 ∼0.4 GB memory on all datasets. The reasons are the efficiency of fuzzy de Bruijn graphs used in Wtdbg2 on the one hand, but also the fact that Wtdbg2 generates consensus sequence in all cases, which corresponds to procedures that are much faster than procedures that address strain aware assembly. The second fastest tool is CliqueSNV, which, however, is reference based, which puts direct comparisons into context. For simulated PacBio CLR reads assembly, Strainline is 1.4 ∼16 times faster and requires less or similar peak memory in comparison to Canu (Additional file 1: Table S6). For simulated ONT reads assembly, Strainline is 15∼76 times faster and requires less or similar peak memory in comparison to Canu (Additional file 1: Table S7). By design, the most expensive steps of Strainline are threaded, and there are two steps that consume time, namely ‘Correction’ and ‘Consensus’, see Fig. 1. Overall, Strainline requires 12 to 177 CPU hours and 15 to 45 GB main memory on the datasets of 20,000 × coverage. This indicates that our method is very well applicable in all real world scenarios of interest.

## Discussion

We have presented Strainline, a de novo assembly approach that reconstructs haplotype-specific genomes from mixed viral samples using noisy long-read sequencing data. To the best of our knowledge, Strainline is the first such approach that is presented in the literature.

Although the length of long reads is a major advantage in the assembly of genomes, the greatly elevated error rates pose substantial challenges when seeking to distinguish between little diverse genomes. The large number of sequencing errors that affects the reads easily exceeds the amount of genetic variants that are characteristic of the different genomes. Because co-occurring true mutations can mean a decisive handle in the differential analysis, it is usually advantageous to make use of the reads at their full length. However, addressing this particular challenge by computing all-vs-all overlaps of reads may demand excessive runtimes.

To address these major challenges, we proceed by drawing from both de Bruijn graph based and overlap graph-based techniques so as to combine the virtues of the two paradigms. We first employ a local de Bruijn graph-based strategy by way of an initial error correction step. Remarkably, this strategy was originally been designed and presented for correcting long reads sampled from prokaryotic and eukaryotic genomes, without that the local de Bruijn graph-based strategy established the state of the art on such longer genomes. Here, we realized that local de Bruijn graphs perform perfectly fine when processing short genomes: sequencing errors are suppressed succesfully which enables us to carry out the second step.

The second step clusters the pre-corrected long reads into groups of reads that are supposed to collect reads from identical haplotypes. To avoid excessive overlap computations, we iteratively select seed reads, as longest reads that do not overlap any of the previously selected seed reads. If reads sufficiently overlap a seed read, we put them into the cluster of the respective seed read. This way, we determine clusters based on seed-vs-all overlap computations. Because the number of seed reads is smaller by orders of magnitude in comparison with the number of reads overall, we reduce the runtime by orders of magnitude in comparison with performing all-vs-all overlap computations.

Following cluster generation, we compute a haplotype-specific consensus (haplotig) for each cluster of reads. This eliminates errors further and preserves haplotype-specific variation. Upon having computed this consensus, we iteratively extend the haplotigs by evaluating their overlaps—note that the number of haplotigs is much smaller than the number of reads, such that all-vs-all haplotig overlap computations are computationally feasible. In a last step, we discard haplotigs (haplotypes) of too low divergence or abundance. The final output is a set of haplotypes together with their abundances. Many of the haplotypes have reached full length and are reliable in terms of the sequence content. In this, the goal of *de novo viral quasispecies assembly from noisy long reads has been achieved*.

Benchmarking experiments on both simulated and experimental data, reflecting various mixed viral samples referring to various relevant settings, such as different viruses, different numbers of strains, haplotype abundances and sequencing platforms (PacBio/ONT), have shown that our approach accurately reconstructs the haplotype-specific sequences. Thereby, the output contigs tend to cover the majority of haplotypes at their full length on most data sets.

As a result, Strainline proves superior in comparison with all long read genome assembly methods currently available, and in theory applicable for virus genomes; note again that no alternative method explicitly addresses virus genomes. The superiority of Strainline becomes documented in terms of various well-known and -approved assembly evaluation metrics: Strainline’s contigs cover substantially more haplotypes, are longer (N50, NGA50) and are more accurate in terms of error and misassembly rates.

Clearly, Strainline’s superiority did not come as a particular surprise, as representing the first approach to explicitly consider de novo virus genome assembly from long reads. In some detail, Canu and CliqueSNV at least are able to recover a certain fraction of haplotypes, whereas Wtdbg2, however, always outputs one consensus sequence. With Canu, CliqueSNV and Wtdbg2 as the only approaches available at all, Strainline arguably establishes a substantial step up in the haplotype-specific assembly of viral genomes from noisy third-generation sequencing reads.

There were some further clear hints that our approach made sense. First, we demonstrated that Strainline was able to exploit increasing sequencing coverage to its advantage; note that deeply sequenced datasets are common when analyzing virus genomes. At the same time, Strainline required the least amount of reads for establishing sufficiently accurate haplotypes in comparison with other methods. This indicates that Strainline caters to a greater range of experimental settings of practical interest.

In addition, Strainline is the only long-read de novo assembly approach available that does not only assemble the viral genomes, but also estimates the abundances of the haplotypes that make part of the mix of viral strains. Results have demonstrated that Strainline’s abundance estimates are sufficiently accurate if the abundances do not refer to strains of very low relative frequencies. Note however that low-frequency strains pose particular challenges not only in this respect, because the relative lack of coverage for such strains raises the level of uncertainty one has to deal with.

Note finally that in comparison with short-read viral quasispecies assemblers (such as most prominently [18, 19]) that accurately operate in a haplotype-specific way, Strainline is the only approach that reconstructs the haplotypes for most datasets at their full length. This does not only point out that long reads indeed do mean a major advantage over short reads, but also means that Strainline is able to leverage the advantages of long reads successfully.

Nonetheless, improvements are conceivable. Strainline struggles to reconstruct very low abundant haplotypes such as 0.01% or 0.1% (at least if divergence of strains is low) when the overall sequencing coverage is 20,000×, so there is currently no method available to successfully reconstruct such haplotypes.

Also, Strainline sometimes tends to overestimate the number of haplotypes, which, as a consequence, hampers the estimation of the abundances of the strains. One possible reason for haplotype overestimation is that haplotype identity in read overlaps is based on overlap length and sequence identity alone, which may be too simplistic. Likely, more sophisticated criteria will be able to successfully address this issue, which we consider valuable future work. Last but not least, the computational efficiency of the approach likely leaves further room for improvements: for example, computation of consensus sequence for read clusters can possibly be implemented in a more efficient way.

## Conclusions

This paper presents Strainline, an approach to full-length viral haplotype reconstruction from noisy long-read sequencing data. Strainline operates de novo, that is, Strainline does not make use of reference sequence any time. We make use of local de Bruijn graph assembly to sufficiently correct sequencing errors in raw reads, such that it is possible to extend contigs iteratively at a haplotype-specific level, in order to eventually yield full-length strain-specific haplotypes. These properties render Strainline unique in the spectrum of currently available assemblers: it is the only approach that can reconstruct the strain-specific haplotypes in mixed viral samples using long reads, and estimate their abundances sufficiently accurately.

We remain with saying that databases (e.g., GISAID) are currently filling up with (in particular SARS-CoV-2) TGS sequencing read samples, drawn from infected people. So far, one has been blind with respect to counting the number of strains that commonly affect their hosts without incurring reference induced biases. The biased view on the amount of strains that have infected people, either initially or having formed during the course of the infection, is a decisive hindrance when assessing the evolutionary development of viruses, where SARS-CoV-2 is an example of particular current interest. Seeing the full spectrum of strains, without having to make use of existing, potentially already obsolete reference genomes, has the potential to yield major insight into the course of epidemics.

Now, we can finally have a closer look.

Our approach is implemented in an easy-to-use open-source tool https://github.com/HaploKit/Strainline.

## Methods

### Correcting sequencing errors

For initial correction of errors in long reads, we adopt a local de Bruijn graph assembly based strategy. While de Bruijn graph-based data structures tend to have difficulties when dealing with TGS data because of the high error rates, it is shown in [27] that it can work effectively when applied to small segments of the long reads.

Here, we realized that the corresponding strategy is particularly powerful when applied to virus TGS data. In our experiments, we observed that the local de Bruijn graph-based strategy has substantial advantages on virus data in comparison to the results presented in the seminal work [27], which exclusively focused on TGS data from prokaryotic and eukaryotic genomes of length at least a few Mbp. We integrate Daccord, as originally suggested [27], by straightforwardly adjusting parameters so as to account for particularities of datasets under consideration here, but without any methodical or theoretical adjustments, into our workflow.

The main steps of the workflow that address error correction are shown in Fig. 2. In a first step (see “Target read & overlapping reads”), read overlaps of raw reads are computed using Daligner V2.0 [37], which uses canonical k-mers to identify local alignments of high confidence between reads. Upon having selected a target read (i.e. the read to be corrected), we subsequently (see “Read alignment pile”) form a read alignment pile, consisting of the target read and all reads that share significant overlap with it. Then (see “Windows”), we divide the pile into small windows, which serves the application of the local de Bruijn graph-based strategy; note that the windows have to be sufficiently small (here: 40 bp) such that the strategy works satisfyingly.

Accordingly, we construct de Bruijn graphs for all windows of size 40 bp (see “DBGs for all windows” in Fig. 2). Importantly, windows share an overlapping interval of 30 bp (that is, the step size of a window is 10bp, which is controlled by the parameter -a: advance size in Daccord), as one can see in “Window consensus”: windows and their overlapping intervals can be interpreted as nodes and edges of another graph. The respective graph of windows and overlapping intervals can then be traversed, where scores can be assigned to paths through that graph. To be specific, a path *v*_1_,*v*_2_,...,*v*_*n*_ through a local de Bruijn graph is assigned the score $ {\textstyle \sum _{i=1}^{n} \text {kscore}(v_{i},i-1)} $, where *v*_*i*_ is the node (i.e. a k-mer) in the graph, and kscore(*v*_*i*_,*i*−1) is the function for scoring a pair consisting of a k-mer *v*_*i*_ and a position *i*−1 the k-mer may occur (see [27] for more details).

The paths of windows through that graph that are optimal in terms of the scores are determined, and are further evaluated with respect to differences with the target read; the concatenation of sequences of the graph that has highest score and least differences in comparison with the target read is taken as the consensus sequence (corresponds to “Window consensus” in Fig. 2), and reflects the true sequence that underlies the target read. One can then correct the errors in the target read accordingly.

### Read cluster generation

Subsequently, we compute clusters of (error corrected) overlapping reads. This addresses to wipe out further errors, and, as the major point, to form groups of reads all of which stem from the same (local) haplotype. For pseudo code supporting the generation of read clusters, see Algorithm 1.

For this, first, we sort the error corrected reads by length in decreasing order, considering that longer reads tend to have more overlaps. Processing reads in the corresponding order therefore results in larger read clusters. This increases the length of the resulting haplotigs and hence improves the assembly overall. In each iteration, we choose the longest read having remained not assigned to a cluster as the seed read and compute seed-vs-all overlaps on corrected reads using Minimap2 [38], whose seed-chain-align procedure is known to perform pairwise alignments extremely fast.

Bad overlaps are filtered out according to reasonable, additional criteria, such as minimum read overlap length (–minOvlpLen), minimum level of sequence identity for read overlaps (–minIdentity), and in case of self-overlaps, duplicates or internal matches (maximum overhang length of overlaps, –maxOH). Overlaps that do not satisfy these criteria are removed.

To define these criteria concretely, we follow Algorithm 5 in [39]. Enforced by choosing relatively strict thresholds for these criteria (see [40] for the details), the remaining overlapping reads are expected to stem from the haplotype of the seed read. The corresponding cluster is determined as the set of reads that overlap the seed read (according to the criteria listed above).

Subsequently, all of the reads of the cluster are discarded from the sorted list of reads, and the next iteration (referring to line 5 in Algorithm 1) is executed. The procedure stops when the number of iterations (hence clusters) reaches the upper limit *k* where *k* is user defined (default 100), or all reads have been processed (corresponding to *R*=*∅* in Algorithm 1).

Notably, we only compute seed-vs-all overlaps, and not all-vs-all overlaps (as per, for example, a straightforward, naive approach), and limit the number of clusters, which decisively speeds up the procedure. In this, the fact that reads are already error corrected ensures that one does not miss any overlap, which overall prevents to compute all-vs-all overlaps.


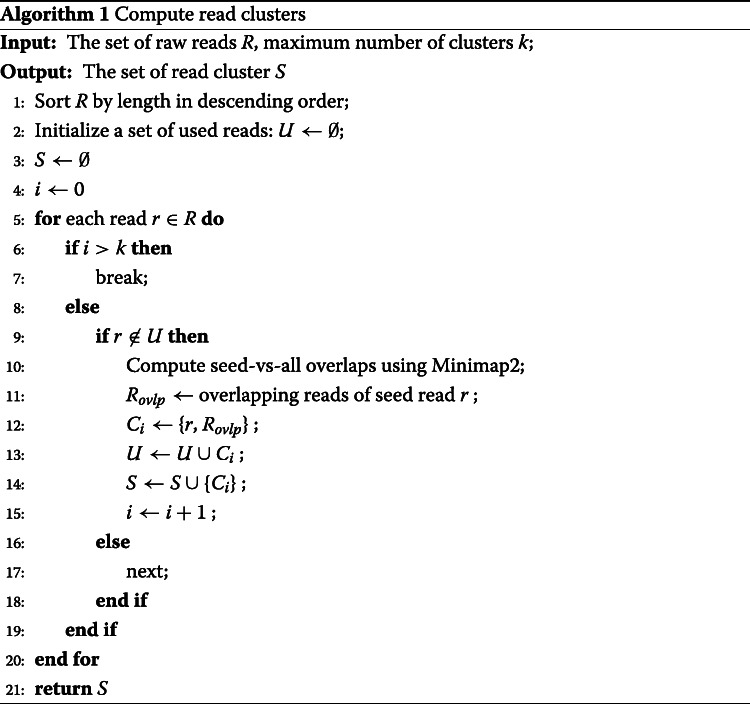


### Generating a consensus sequence for each read cluster

Although reads were initially corrected, they may still contain errors. The major possible reasons are near-identical genomic regions that are shared across haplotypes.

For final polishing of reads, and removal of also more stubbornly resisting errors, we first compute a partial order alignment (POA) algorithm [41] for each cluster. Subsequently, we generate the consensus sequence for the POA of each cluster (which is a straightforward, generic procedure). Adapting reads to this consensus completes the process of error correction.

For computing POA’s of clusters, we make use of the fast SIMD version, as implemented in Spoa [34], and built into our approach. Note that this step can generate a longer and more accurate haplotype-specific sequence for each cluster (which reflects the cluster-specific haplotig).

### Iterative extension of haplotigs

Haplotigs generated from the previous step do not necessarily reflect full-length haplotypes. This explains why it makes sense to try to extend them further.

For extending haplotigs, one considers haplotigs as reads, and re-runs “Read cluster generation” and “Generating a consensus sequence for each read cluster” another time, inserting the haplotigs of the first iteration as reads. The procedure is iteratively repeated until haplotigs cannot be elongated further. In that, our experiments demonstrate that two iterations usually suffice for virus sequencing data sets.

### Haplotype filtration

Ideally, iteratively extending haplotigs eventually results in correct, full-length haplotypes. However, in practice, it is possible that some haplotypes have very low pairwise divergence or very low relative abundance, each of which indicates that the corresponding haplotypes are likely to reflect artifacts. It is therefore reasonable to filter out such artificial haplotypes, because they either reflect redundant or spurious sequences. For the identification of low divergence and low relative abundance haplotypes, we make use of two procedures for computing haplotype divergence on the one hand, and haplotype relative abundance on the other hand.

**Haplotype divergence calculation.** We propose two metrics for haplotype divergence measurement, namely local divergence (LD) and global divergence (GD). Given two haplotypes *H*_*i*_,*H*_*j*_, let *l* be the length of their overlap, and *m* be the number of identically matching positions in the overlap (so *m*≤*l* by definition of *l*,*m*). Let further *n*_*i*_,*n*_*j*_ be the lengths of the non-overlapping parts of *H*_*i*_,*H*_*j*_ relative to their overlap.

LD is defined by the formula *LD*(*H*_*i*_,*H*_*j*_)=1−*m*/*l* and GD is defined by *GD*(*H*_*i*_,*H*_*j*_)=1−*m*/(*l*+*n*_*i*_+*n*_*j*_). In other words, LD agrees with BLAST-like alignment identity, when only considering the overlapping regions. GD, on the other hand, considers the entire sequence context that neighbors and includes the overlap of *H*_*i*_ and *H*_*j*_.

Note that two haplotypes having low local divergence but large global divergence (because of a long overhang) are more likely to stem from two different strains than haplotypes having small LD and GD.

Let further *maxLD* and *maxCO* represent the user-defined maximum local divergence and the maximum contig overhang length (5bp in our cases), respectively. Note that *H*_*i*_ being contained in *H*_*j*_ implies the length of *H*_*i*_ being smaller than the length of *H*_*j*_, as well as *LD*(*H*_*i*_,*H*_*j*_<*maxLD* and contig overhang length being at most *maxCO*. In this case, *H*_*i*_ is discarded from the downstream analysis. For determining the overlap information of two haplotypes, Minimap2 is used. The ultimate output is a set of non-redundant haplotypes.

**Calculating relative abundance of haplotypes.** Calculating haplotype relative abundance is straightforward when the length of the haplotypes approaches the size of the (strain-specific) genomes, and when original reads are nearly free of errors.

For the calculation, one aligns the reads with the haplotypes, which in the given situation can usually be done in a non-ambiguous way. The result of the alignments is stored in a BAM file. We then adopt the jgi_summarize_bam_contig_depths program from MetaBAT 2 [42] to calculate the depth of haplotypes based on the BAM file. The relative abundance of *H*_*i*_ is equal to the average depth of *H*_*i*_ divided by the overall average depth of all haplotypes. Haplotypes with very low relative abundance are filtered out, and one recomputes the abundance for the remaining haplotypes upon removal of the spurious, low abundance haplotypes.

The final output consists of a set of full-length haplotypes along with their corresponding relative frequencies, as desired.

### Data simulation

To evaluate performance of Strainline, we generated several simulated datasets for both PacBio CLR and ONT reads. For simulating reads, we made use of PBSIM V1.0.3 [28] model-based simulation, which reflects a sound way to generate PacBio CLR reads of N50 length 2.4kbp and average sequencing error rate 10%. In addition, we also downloaded real Oxford Nanopore reads (GridION) of a SARS-CoV-2 sample from the SRA database (SRP250446) and then used NanoSim V2.6.0 [29] as a popular, approved simulator to train an ONT read profile based on this real ONT dataset. Accordingly, we generated simulated ONT reads, at an N50 of 2.5kbp in terms of length and average sequencing error rate of 10%. The genomes used for each dataset are listed in the “Availability of data and materials” section.

### Parameter settings

Three main parameters need to be set when running Strainline. The first parameter is *k*, the maximum number of clusters in Algorithm 1. To investigate the consequences of varying *k*, we ran Strainline with *k* ranging from 40 to 200 on the 6-strain Poliovirus mixture (PacBio CLR reads). Results is shown in Additional file 1: Table S8; they show that optimal, robust assembly performance is reached for *k* ranging between 60 and 200. While using even larger *k* may improve the assembly performance slightly further, choices beyond 200 require computational resources that are too demanding.

Based on these analyses, we recommend users to choose *k* between 50 and 200, where a typical choice is *k*=100, as used in the majority of our experiments. The other two parameters are maximum global divergence (*maxGD*) and maximum local divergence (*maxLD*) as described in the “Haplotype filtration” section. Usually, the amount of haplotypes reconstructed increases on lowering *maxGD* or *maxLD*, so increases recall. However, this advantage is offset by an overestimation of haplotypes, which lowers precision. Here, we chose maxGD = 0.02, maxLD = 0.01 for most of our data sets. Detailed settings of parameters used in our experiments can be seen at Code Ocean [40].

## Supplementary Information

**Additional file 1** Supplement: This contains all supplementary materials referenced in the main text.

**Additional file 2** Review history.

## Data Availability

All data (including raw sequencing reads, reference genomes and assemblies) and code for reproducing the results in the paper are deposited on Code Ocean [40]. The source code of Strainline is GPL-3.0 licensed, and publicly available on GitHub [43].

## Footnotes

1 Receiving the error message “No disjointigs were assembled”; upon contact, the authors responded that metaFlye does not support the assembly of very short sequences, such as viruses.
