# Supplementary material for: Strainline: full-length de novo viral haplotype reconstruction from noisy long reads
Source: Genome Biol. 2022 Jan 20;23:29. doi: 10.1186/s13059-021-02587-6 (PMC8771625; doi:10.1186/s13059-021-02587-6)
Supplement: Supplementary file 1 — Additional file 1 Supplement: This contains all supplementary materials referenced in the main text. [file 13059_2021_2587_MOESM1_ESM.pdf]

Supplementary Material
Strainline: full-length de novo viral haplotype reconstruction from noisy long reads

Xiao Luo[1,2,†], Xiongbin Kang[1,2,†], Alexander Schönhuth[1,2,*]

[1] Centrum Wiskunde & Informatica, Amsterdam, The Netherlands
[2] Genome Data Science, Faculty of Technology, Bielefeld University, Bielefeld, Germany

[†]These authors contributed equally to the work.
[*]To whom correspondence should be addressed.

(a)

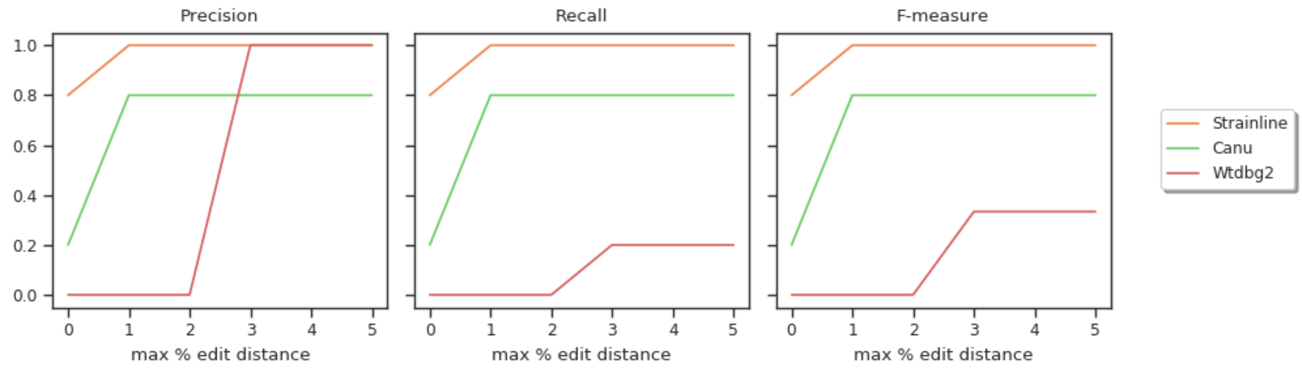
Precision
Recall
F-measure
1.0
0.8
0.6
0.4
0.2
0.0
0
1
2
3
4
5
max % edit distance
Strainline
Canu
Wtdbg2

(b)

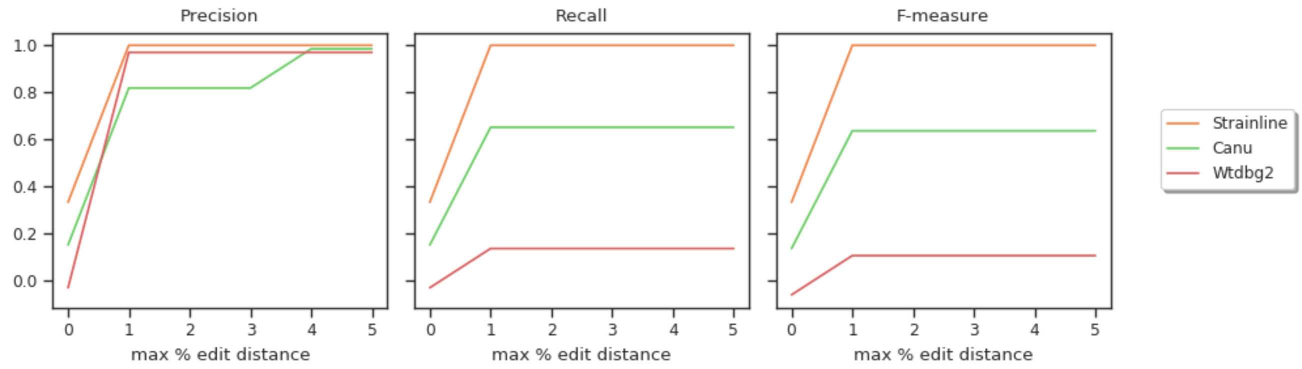
Precision
Recall
F-measure
1.0
0.8
0.6
0.4
0.2
0.0
0
1
2
3
4
5
max % edit distance
Strainline
Canu
Wtdbg2

(c)

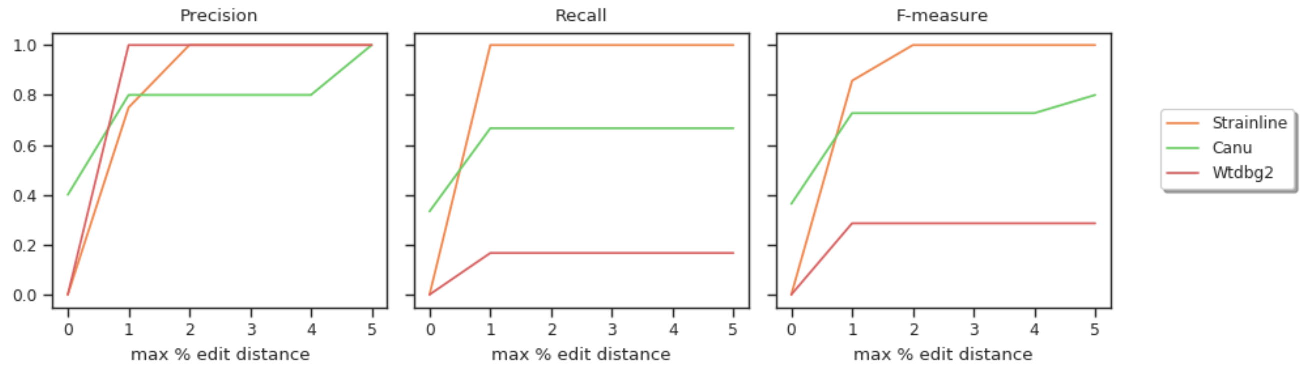
Precision
Recall
F-measure
1.0
0.8
0.6
0.4
0.2
0.0
0
1
2
3
4
5
max % edit distance
Strainline
Canu
Wtdbg2

(d)

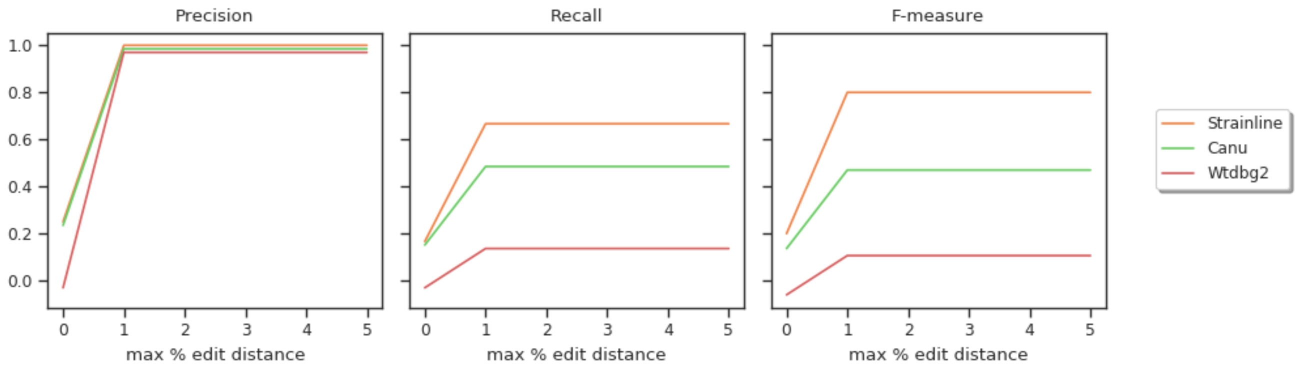
Precision
Recall
F-measure
1.0
0.8
0.6
0.4
0.2
0.0
0
1
2
3
4
5
max % edit distance
Strainline
Canu
Wtdbg2

(e)

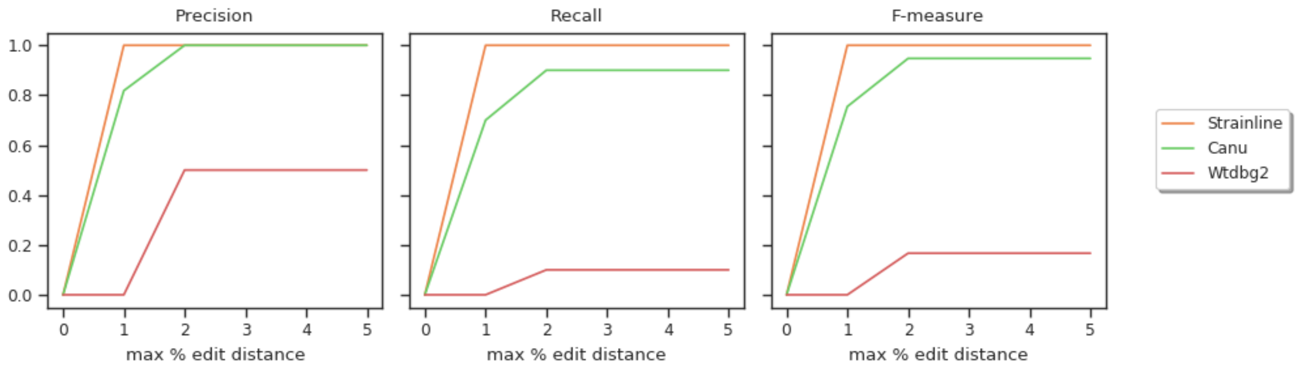
Precision
Recall
F-measure
1.0
0.8
0.6
0.4
0.2
0.0
0
1
2
3
4
5
max % edit distance
Strainline
Canu
Wtdbg2

(f)

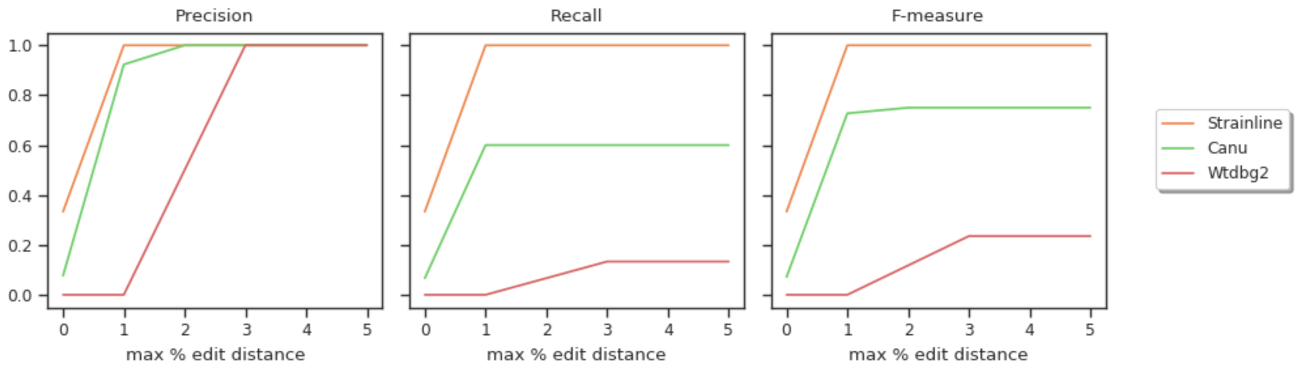
Precision
Recall
F-measure
1.0
0.8
0.6
0.4
0.2
0.0
0
1
2
3
4
5
max % edit distance
Strainline
Canu
Wtdbg2

(g)

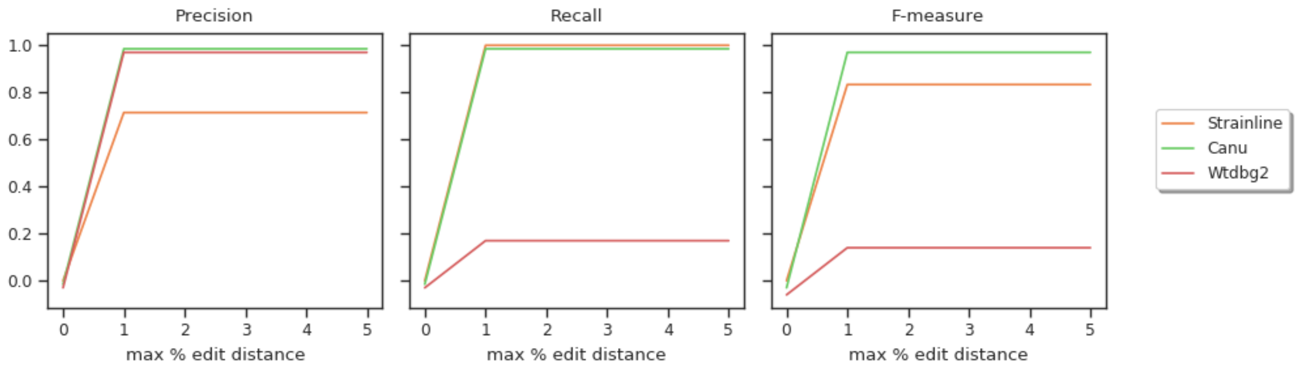

(h)

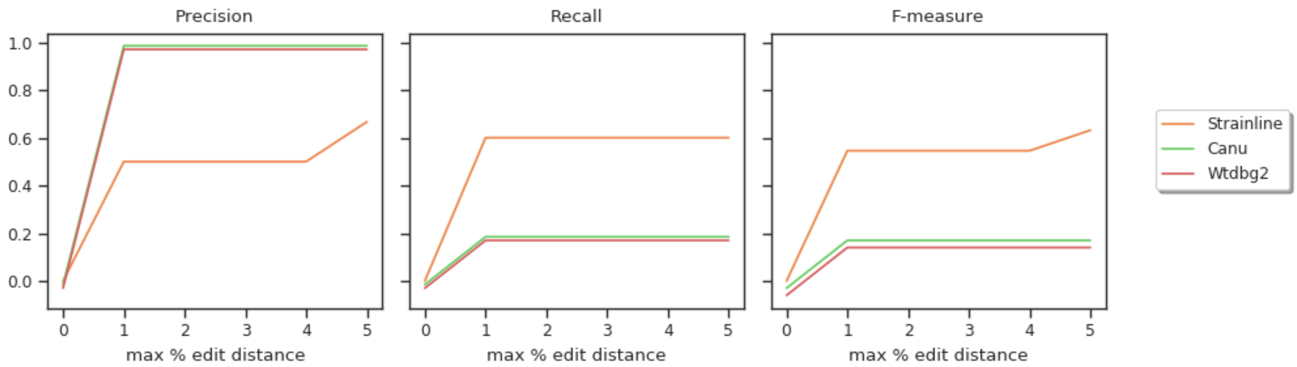

Figure S1. Precision, recall and F-measure for different viral mixtures (simulated PacBio CLR reads). **a,b,c,d,e,f,g,h** represent 5-strain HIV, 6-strain Poliovirus, 6-strain Poliovirus (la1), 6-strain Poliovirus (la2), 10-strain HCV, 15-strain ZIKV, 5-strain SARS-CoV-2, 5-strain SARS-CoV-2 (la) mixtures, respectively. The x-axis denotes the various thresholds (max edit distance) which are used to determine if an assembled contig is assigned to a true strain.

(a)

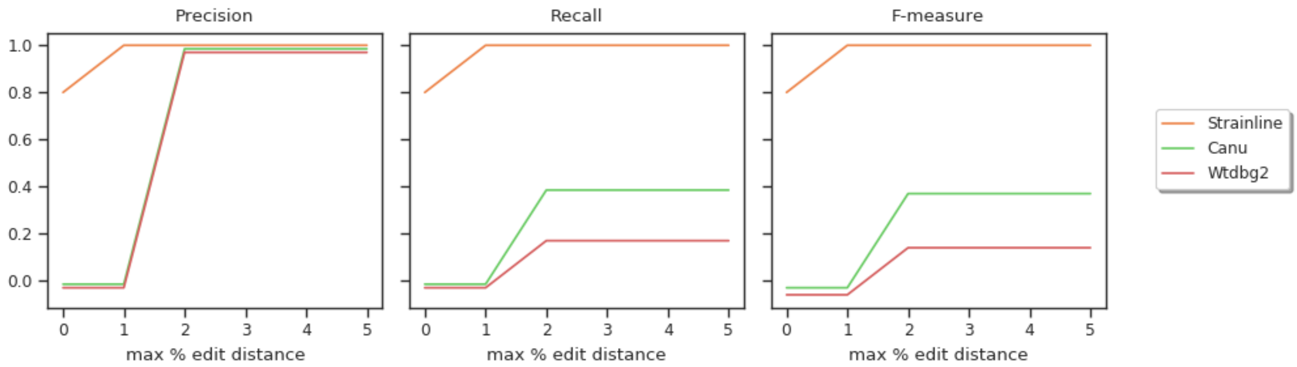
Precision
Recall
F-measure
1.0
0.8
0.6
0.4
0.2
0.0
0 1 2 3 4 5
max % edit distance
Strainline
Canu
Wtdbg2

(b)

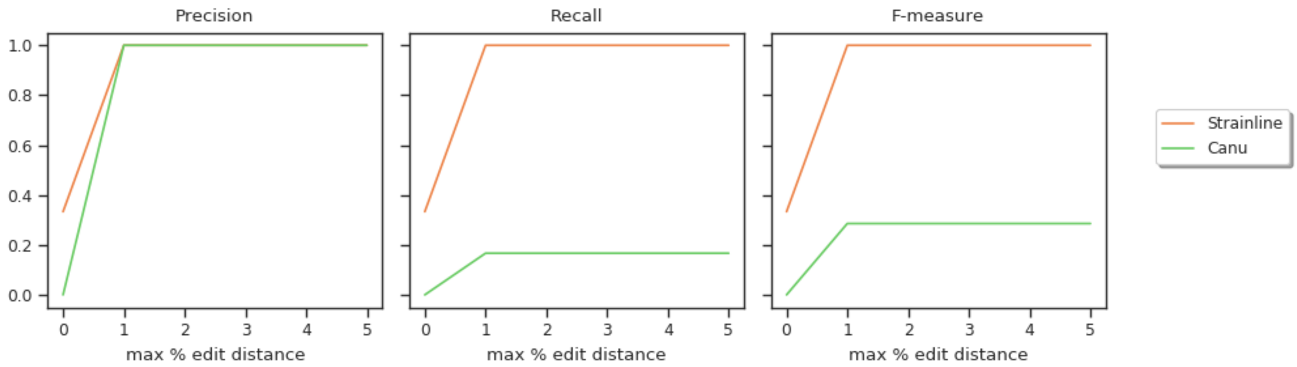
Precision
Recall
F-measure
1.0
0.8
0.6
0.4
0.2
0.0
0 1 2 3 4 5
max % edit distance
Strainline
Canu

(c)

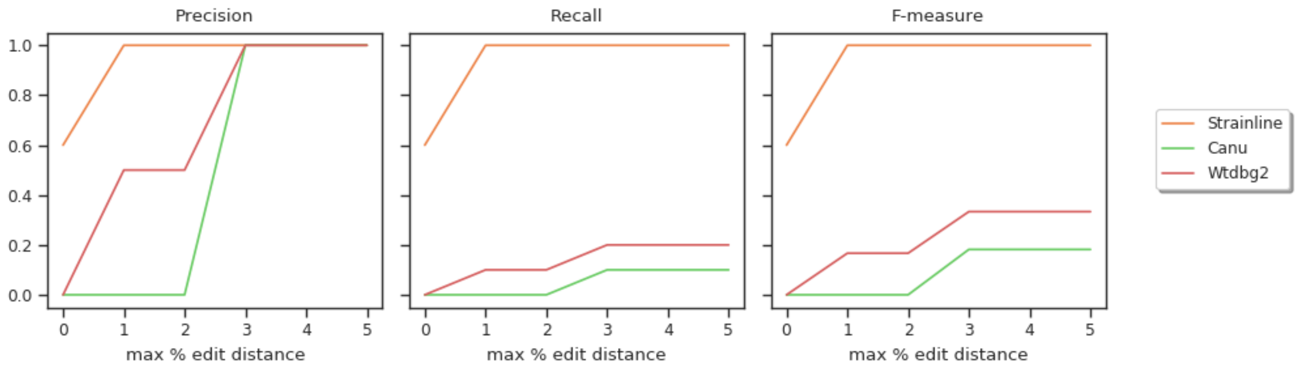
Precision
Recall
F-measure
1.0
0.8
0.6
0.4
0.2
0.0
0 1 2 3 4 5
max % edit distance
Strainline
Canu
Wtdbg2

(d)

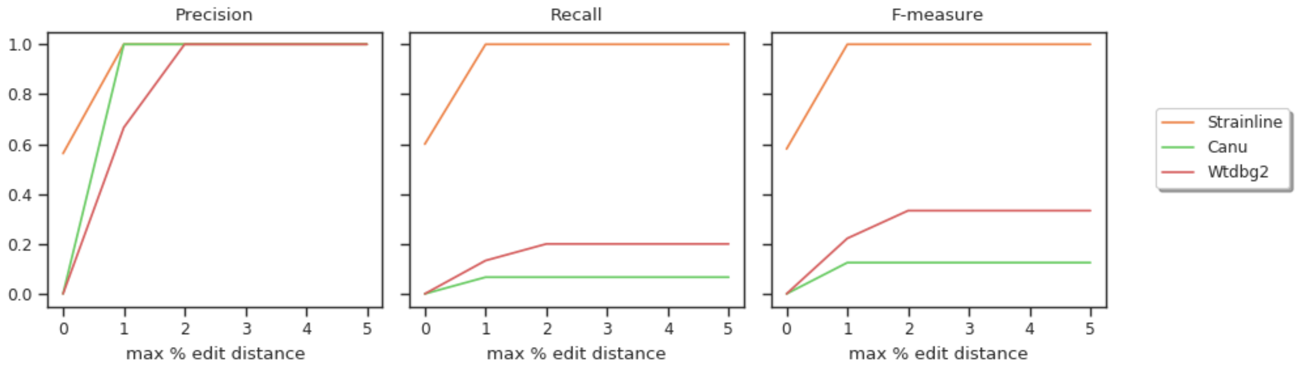

(e)

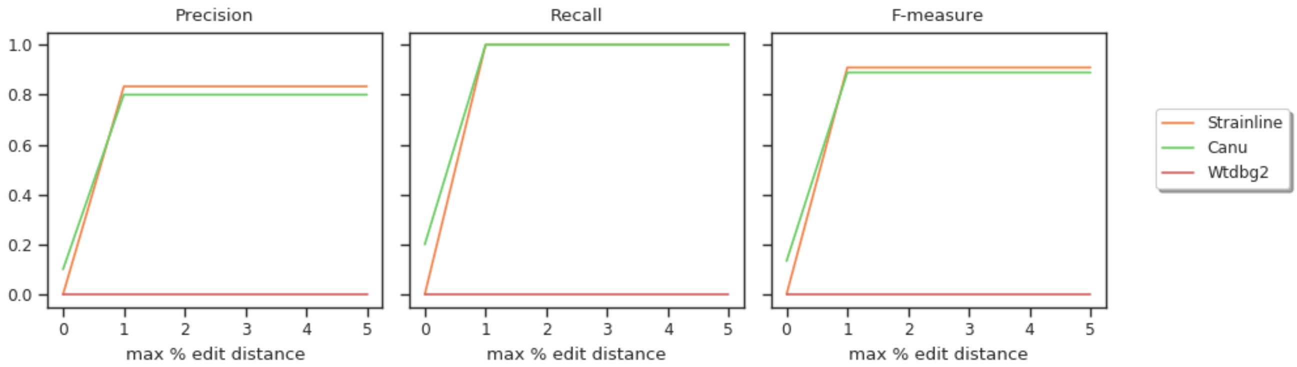

Figure S2. Precision, recall and F-measure for different viral mixtures (simulated Oxford Nanopore reads). **a,b,c,d,e** represent 5-strain HIV, 6-strain Poliovirus, 10-strain HCV, 15-strain ZIKV, 5-strain SARS-CoV-2 mixtures, respectively. The x-axis denotes the various thresholds (max edit distance) which are used to determine if an assembled contig is assigned to a true strain.

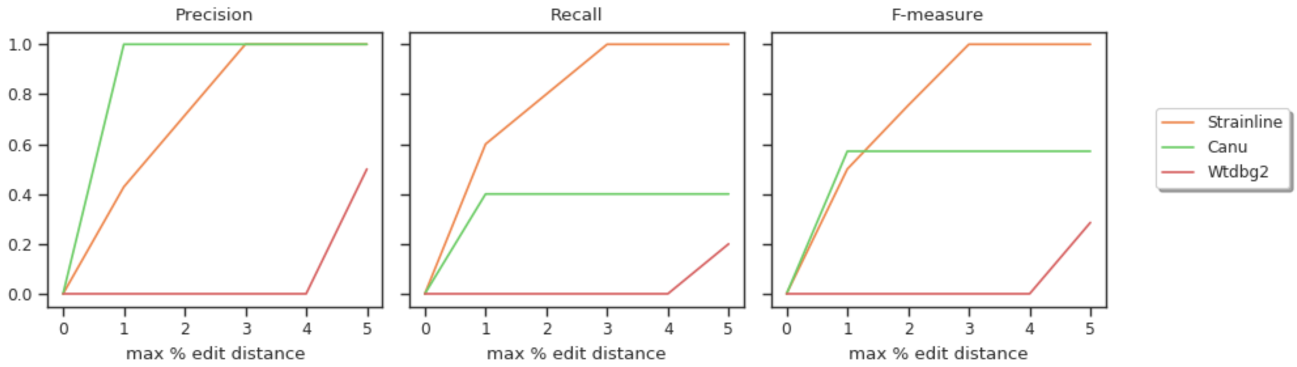

Figure S3. Precision, recall and F-measure for 5-strain PVY mixture (real data). The x-axis denotes the various thresholds (max edit distance) which are used to determine if an assembled contig is assigned to a true strain.

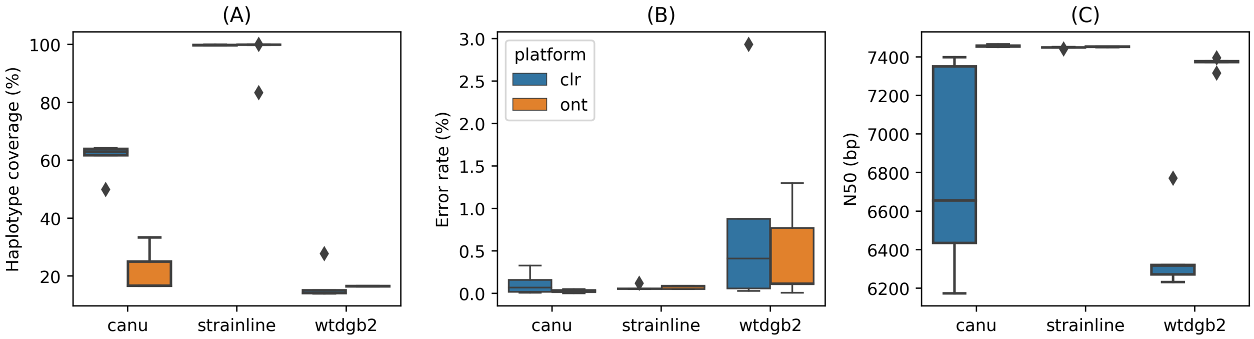

Figure S4. Assembly performance for repeated benchmarks. We repeatedly simulated another five *6-strain poliovirus mixture* data sets for both PacBio CLR and ONT reads, and then ran Strainline, Canu, Wtdbg2 for comparison while keeping the identical parameters in each data set. The left (blue) and the right (orange) boxplots represent the results of PacBio CLR and ONT reads, respectively. (A), (B) and (C) show the results of haplotype coverage, error rate and N50, respectively.

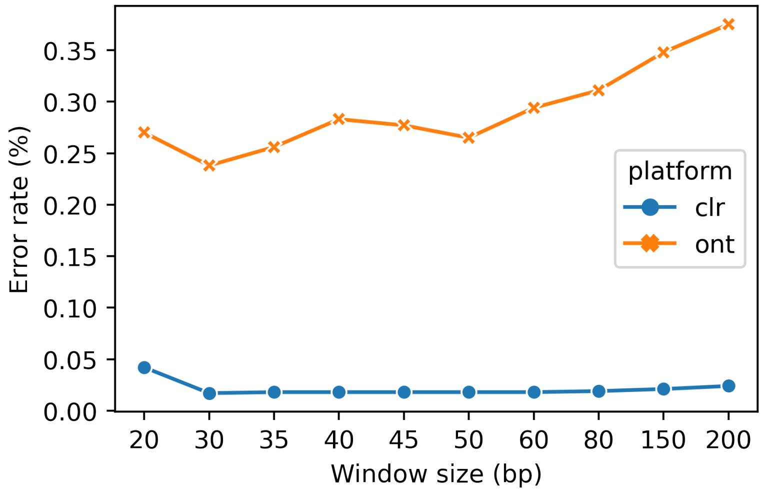

Figure S5. Error correction performances with different window size. We tested the performances of Strainline's error correction step (Daccord) using window size from 20 to 200. The blue and orange lines represent the results of PacBio CLR and ONT data from *5-strain HIV mixture*, respectively.

Virus mixture	Accession number of the ground truth genomes
Simulated	
5-strain HIV	896, HXB2, JRCSF, NL43, YU2
6-strain Poliovirus	MG212491.1, MG212490.1, MG212489.1<br>MG212484.1, MG212475.1, MG212469.1
10-strain HCV	EU155339.2, EU155344.2, EU234065.2, EU255965.1, EU255973.1<br>EU255980.1, EU255981.1, EU255982.1, EU255983.1, EU255989.1
15-strain ZIKV	HQ234500.1, HQ234501.1, HQ234498.1
5-strain SARS-CoV-2	Egypt/MASRI-C5-020/2020, USA/OH-OSUP0014/2020,<br>Oman/89477/2020, Belgium/UZA-UA-TIQ235/2021,<br>Wuhan-Hu-1(NC_045512.2)
Experimental	
5-strain PVY (Mock)	MT264731.1, MT264732.1, MT264733.1, MT264735.1, MT264734.1
SARS-CoV-2 (Real sample)	-

Table S1. Source of genomes used in benchmarking data sets. Genomes in 5-strain HIV mixture were presented as a gold standard benchmark in (Giallonardo, et al., 2014) and are available at `https://github.com/cbg-ethz/5-virus-mix`. Genomes in 5-strain SARS-CoV-2 mixture are from the GISAID database except Wuhan-Hu-1 is from NCBI. Other genomes are from NCBI.

	#Contigs	HC (%)	N50 (bp)	NGA50 (bp)	ER (%)	MC(%)
5-strain HIV mixture						
CliqueSNV-h-ref-raw	-	-	-	-	-	-
CliqueSNV-h-ref-corr	1	20.1	9711	-	3.275	0.0
CliqueSNV-b-ref-raw (Wtdbg2)	4	46.3	7419	7411	1.062	0.0
CliqueSNV-b-ref-corr (Wtdbg2)	8	77.2	7419	7419	1.106	0.0
6-strain Poliovirus mixture						
CliqueSNV-h-ref-corr	4	49.9	7452	7438	0.433	0.0
CliqueSNV-b-ref-corr (Wtdbg2)	2	29.3	6575	-	0.259	0.0
10-strain HCV mixture						
CliqueSNV-h-ref-corr	1	10.0	9310	-	1.963	0.0
CliqueSNV-b-ref-corr (Wtdbg2)	-	-	-	-	-	-
15-strain ZIKV mixture						
CliqueSNV-h-ref-corr	1	6.7	10268	-	1.627	0.0
CliqueSNV-b-ref-corr (Wtdbg2)	-	-	-	-	-	-
5-strain SARS-CoV-2 mixture						
CliqueSNV-h-ref-corr	1	21.1	29903	-	0.007	0.0
CliqueSNV-b-ref-corr (Wtdbg2)	7	20.6	29151	29134	0.255	0.0

Table S2. Benchmarking results of reference-guided method CliqueSNV for simulated PacBio CLR reads. We present results of CliqueSNV using different strategies, such as using the highest abundant reference genome (the ground truth, denoted as h-ref) as well as an ad hoc, bootstrap reference genome (b-ref) generated by Wtdbg2. For 5-strain HIV mixture, we also present results of CliqueSNV using raw reads (-raw) and error corrected reads (-corr) generated by Strainline error correction. For other data sets except 5-strain HIV mixture, we only report the results of using corrected reads because of better performance. The results fully filled with '-' indicate that the program returns error when parsing text SAM file on this dataset.

	#Contigs*	HC (%)	N50 (bp)	NGA50 (bp)	ER (%)	MC(%)
6-strain Poliovirus (la1) mixture						
Strainline	8	98.0	7402	7395	0.300	0.0
Canu	5	59.0	7332	4965	0.769	0.0
Wtdbg2	1	14.3	6391	-	0.000	0.0
CliqueSNV-h-ref-corr	2	33.3	7452	-	0.013	0.0
6-strain Poliovirus (la2) mixture						
Strainline	4	65.3	7417	6938	0.271	0.0
Canu	4	49.0	7437	5752	0.098	0.0
Wtdbg2	1	15.5	6943	-	0.058	0.0
CliqueSNV-h-ref-corr	1	16.7	7453	-	0.000	0.0
CliqueSNV-b-ref-corr (Strainline)	1	16.7	7450	-	0.000	0.0
5-strain SARS-CoV-2 (la) mixture						
Strainline	6	60.8	29432	27307	0.055	0.0
Canu	-	-	-	-	-	-
Wtdbg2	1	20.2	28569	-	0.056	0.0
CliqueSNV-h-ref-corr	4	21.1	29903	-	0.005	0.0
CliqueSNV-b-ref-corr (Strainline)	-	-	-	-	-	-

Table S3. Benchmarking results for three additionally simulated datasets with very low abundant (la) strains. The total sequencing coverage in this table is 20000×. In 6-strain Poliovirus (la2) and 5-strain SARS-CoV-2 (la) mixtures which Strainline failed to reconstruct all haplotypes, we show the results of "CliqueSNV-b-ref-corr (Strainline)", which means using the consensus of Strainline as the reference for CliqueSNV and using reads corrected by Strainline error correction. Canu and CliqueSNV-b-ref-corr (Strainline) failed to run on 5-strain SARS-CoV-2 (la) mixture.
* If contigs are full-length, this number represents the estimated number of haplotypes or strains in the virus mixture.

	No. of reads	HC (%)	N50 (bp)	ER (%)	Mismatch(%)	Indel(%)
5-strain HIV mixture						
Daccord	51823	99.9	2902	0.023	0.011	0.011
Canu	96119	100.0	2384	1.167	0.638	0.529
LoRMA	46463	99.9	2777	1.349	0.187	1.162
6-strain poliovirus mixture						
Daccord	39773	99.7	2906	0.070	0.021	0.049
Canu	41703	99.7	2630	1.564	0.280	1.284
LoRMA	39930	99.7	2672	1.269	0.181	1.088
6-strain poliovirus (la1) mixture						
Daccord	39526	99.3	2922	0.084	0.018	0.066
Canu	73817	99.7	2391	0.545	0.155	0.390
LoRMA	42323	98.4	2669	1.226	0.170	1.056
6-strain poliovirus (la2) mixture						
Daccord	39318	96.8	2941	0.123	0.019	0.104
Canu	74203	98.8	2389	0.393	0.082	0.310
LoRMA	44910	96.3	2673	1.163	0.157	1.006
10-strain HCV mixture						
Daccord	49729	99.9	2901	0.056	0.045	0.011
Canu	78537	99.9	2509	1.489	0.595	0.894
LoRMA	35605	99.8	2898	1.385	0.186	1.199
15-strain ZIKV mixture						
Daccord	54968	99.6	2898	0.020	0.009	0.011
Canu	64170	99.8	2720	1.078	0.192	0.886
LoRMA	-	-	-	-	-	-
5-strain SARS-CoV-2 mixture						
Daccord	154040	99.9	2909	0.021	0.010	0.011
Canu	289504	100.0	2361	0.785	0.126	0.658
LoRMA	65586	99.8	2148	1.246	0.175	1.071
5-strain SARS-CoV-2 (la) mixture						
Daccord	160075	97.6	2896	0.020	0.008	0.013
Canu	301563	99.2	2362	0.377	0.054	0.323
LoRMA	152241	97.0	2519	1.253	0.167	1.086

Table S4. Benchmarking results for error correction tools on simulated PacBio CLR data. HC = Haplotype Coverage, ER = Error Rate (mismatches + indels). The total sequencing coverage in this table is 20000×. LoRMA failed to run on 15-strain ZIKV mixture.

	No. of reads	HC (%)	N50 (bp)	ER (%)	Mismatch(%)	Indel(%)
5-strain HIV mixture						
Daccord	87342	99.9	2474	0.284	0.112	0.172
Canu	99552	100.0	2521	1.323	0.710	0.613
LoRMA	41233	100.0	1873	0.513	0.097	0.416
6-strain poliovirus mixture						
Daccord	84313	100.0	2563	0.328	0.093	0.235
Canu	99684	100.0	2474	0.697	0.288	0.409
LoRMA	43367	99.7	1846	0.499	0.103	0.396
10-strain HCV mixture						
Daccord	86350	99.9	2569	0.294	0.085	0.208
Canu	99558	100.0	2528	1.562	1.038	0.524
LoRMA	39267	99.9	1890	0.495	0.093	0.402
15-strain ZIKV mixture						
Daccord	87916	99.9	2525	0.912	0.578	0.334
Canu	98742	100.0	2553	1.662	0.878	0.784
LoRMA	35317	99.9	1859	1.108	0.573	0.535
5-strain SARS-CoV-2 mixture						
Daccord	81331	100.0	2694	0.261	0.069	0.192
Canu	64392	100.0	3172	1.329	0.372	0.956
LoRMA	19697	99.5	1630	0.526	0.106	0.421
5-strain PVY mixture						
Daccord	8041	98.7	2016	0.344	0.091	0.253
Canu	34858	99.9	1199	1.481	0.426	1.055
LoRMA	40839	99.6	1116	0.852	0.210	0.641

Table S5. Benchmarking results for error correction tools on ONT data. HC = Haplotype Coverage, ER = Error Rate (mismatches + indels). Note that the 5-strain PVY mixture is the real ONT data, whereas the others are simulated.

	CPU time (h)	peak memory usage (GB)
5-strain HIV mixture		
Strainline	18.9	20.6
Canu	313.5	49.5
Wtdbg2	0.002	0.4
CliqueSNV-h-ref-corr	101.2	31.3
6-strain poliovirus mixture		
Strainline	39.8	14.9
Canu	239.4	52.8
Wtdbg2	0.001	0.1
CliqueSNV-h-ref-corr	0.8	10.1
6-strain poliovirus (la1) mixture		
Strainline	21.1	14.0
Canu	184.1	42.7
Wtdbg2	0.001	0.1
CliqueSNV-h-ref-corr	0.8	6.0
6-strain poliovirus (la2) mixture		
Strainline	14.3	12.4
Canu	167.1	46.8
Wtdbg2	0.001	0.1
CliqueSNV-h-ref-corr	8.6	25.8
10-strain HCV mixture		
Strainline	52.0	20.0
Canu	311.8	29.4
Wtdbg2	0.002	0.1
CliqueSNV-h-ref-corr	34.3	31.2
15-strain ZIKV mixture		
Strainline	99.7	22.3
Canu	143.8	16.3
Wtdbg2	0.001	0.1
CliqueSNV-h-ref-corr	69.8	24.4
5-strain SARS-CoV-2 mixture		
Strainline	107.7	44.1
Canu	667.3	87.5
Wtdbg2	0.006	0.1
CliqueSNV-h-ref-corr	15.2	17.4
5-strain SARS-CoV-2 (la) mixture		
Strainline	177.1	44.6
Canu	>480.6	106.9
Wtdbg2	0.007	0.1
CliqueSNV-h-ref-corr	5.1	14.3

Table S6. Runtime and memory usage for simulated PacBio CLR reads assembly. The total sequencing coverage in this table is 20000×. Canu did not finish on *5-strain SARS-CoV-2 (la) mixture*.

	CPU time (h)	peak memory usage (GB)
5-strain HIV mixture		
Strainline	11.9	19.4
Canu	906.5	11.3
Wtdbg2	0.001	0.4
6-strain poliovirus mixture		
Strainline	24.1	19.2
Canu	803.1	76.5
Wtdbg2	0.001	0.1
10-strain HCV mixture		
Strainline	17.6	17.9
Canu	848.9	80.4
Wtdbg2	0.001	0.1
15-strain ZIKV mixture		
Strainline	77.0	16.5
Canu	1181.4	95.2
Wtdbg2	0.001	0.1
5-strain SARS-CoV-2 mixture		
Strainline	29.6	18.9
Canu	1134.0	127.4
Wtdbg2	0.005	0.2

Table S7. Runtime and memory usage for simulated ONT reads assembly. The total sequencing coverage in this table is 20000×.

k value	#Contigs	HC (%)	N50	NGA50	ER (%)	MC(%)
40	6	96.4	7435	7423	0.046	0.0
60	6	99.7	7442	7430	0.047	0.0
80	6	99.8	7444	7430	0.049	0.0
100	6	99.8	7444	7430	0.052	0.0
120	6	99.8	7444	7430	0.052	0.0
140	6	99.8	7444	7430	0.052	0.0
160	6	99.8	7444	7430	0.052	0.0
200	6	99.8	7444	7430	0.052	0.0

Table S8. Assembly performances of Strainline when choosing different top k seed reads in Algorithm 1. This table uses PacBio CLR reads of *6-strain poliovirus mixture*. HC = Haplotype Coverage, ER = Error Rate (mismatches + indels + 'N's). MC = Misassembled contigs proportion.

	#Contigs	HC (%)	N50	NGA50	ER (%)	MC(%)
Error rate=5%						
Strainline	5	99.7	9698	9698	0.017	0.0
Canu	5	71.2	8211	8211	0.498	20.0
Wtdbg2	1	18.0	8660	-	1.975	0.0
Error rate=10%						
Strainline	5	99.9	9697	9697	0.002	0.0
Canu	5	84.5	8227	8170	0.409	20.0
Wtdbg2	1	15.5	7419	-	1.820	0.0
Error rate=15%						
Strainline	5	99.7	9687	9687	0.044	0.0
Canu	6	77.2	11173	8559	0.069	50.0
Wtdbg2	2	27.3	7474	-	4.597	0.0
Error rate=20%						
Strainline	5	99.6	9686	9673	0.108	0.0
Canu	7	75.6	7355	7972	0.533	14.3
Wtdbg2	-	-	-	-	-	-
Error rate=30%						
Strainline	5	98.3	9594	9573	0.279	0.0
Canu	68	79.7	3443	6178	1.299	0.0
Wtdbg2	-	-	-	-	-	-

Table S9. Assembly performances for PacBio CLR reads with different sequencing error rate. This table uses simulated PacBio CLR reads of *5-strain HIV mixture*. The total sequencing coverage in this table is 20000×. No contig was generated by Wtdbg2 on data sets with error rate of 20% and 30%.

	#Contigs	HC (%)	N50	NGA50	ER (%)	MC(%)
Error rate=5%						
Strainline	5	99.6	9659	9659	0.054	0.0
Canu	2	32.3	14498	-	1.047	50.0
Wtdbg2	1	18.0	8689	-	2.981	0.0
Error rate=10%						
Strainline	5	99.9	9702	9702	0.081	0.0
Canu	2	35.8	18151	7634	1.730	50.0
Wtdbg2	1	18.9	9046	-	1.327	0.0
Error rate=15%						
Strainline	5	99.7	9687	9681	0.058	0.0
Canu	2	44.0	14910	-	1.814	50.0
Wtdbg2	1	18.2	8764	-	1.355	0.0
Error rate=20%						
Strainline	6	97.6	9520	9520	0.113	0.0
Canu	1	31.5	15162	-	2.328	100.0
Wtdbg2	1	17.6	8426	-	1.417	0.0
Error rate=25%						
Strainline	5	99.0	9647	9608	0.119	0.0
Canu	11	82.9	6410	6957	0.864	9.1
Wtdbg2	-	-	-	-	-	-
Error rate=30%						
Strainline	20	0.3	5429	-	6.870	0.0
Canu	15	64.0	4291	5421	2.256	0.0
Wtdbg2	-	-	-	-	-	-

Table S10. Assembly performances for ONT reads with different sequencing error rate. This table uses simulated ONT reads of *5-strain HIV mixture*. The total sequencing coverage in this table is 20000×. No contig was generated by Wtdbg2 on data sets with error rate of 25% and 30%. Note that to control the sequencing error rate of ONT reads, we used PBSIM2 (Ono, Y., et al., 2021) rather than NanoSim to simulate reads with different error rate (except 10%), because NanoSim is unable to control the error rate.

Commands and versions of tools used for comparison

- Strainline v1.0
  See code and commands in the script `/code/reproduce.sh` used for reproducing the results in the paper on Code Ocean: `https://doi.org/10.24433/CO.3155281.v1`.

- Canu v1.9
  #Recommended mode for metagenome assembly
  ```
  Canu genomeSize=$genomesize minReadLength=500 minOverlapLength=200
  corOutCoverage=10000 corMhapSensitivity=high corMinCoverage=0 redMemory=32
  oeaMemory=32 batMemory=200 -pacbio-raw  $fa
  ```

- Wtdbg2 v2.5-h8b12597
  ```
  wtdbg2 -x css/rs/ont -g $genomesize -i $fa -fo out
  wtpoa-cns -i out.ctg.lay.gz -fo out.ctg.fa
  ```

- CliqueSNV v2.0.2
  # We used the command recommended by the authors via email
  ```
  java -jar clique-snv.jar -m snv-illumina  -tf 0.1 -log -in $samfile
  ```

- PredictHaplo v2.1.4
  ```
  predicthaplo --sam $samfile --reference $ref --have_true_haplotypes 1
  ```

- PBSIM v1.0.3-h2d50403_2
  ```
  pbsim --length-mean 2000 --accuracy-mean 0.9 --length-max 6000
  --length-min 500 --depth $depth --model_qc $model_clr $ref
  ```

- NanoSim v2.6.0
  # training process
  ```
  simulator.py genome -i train_reads.fa -rg train_ref.fa -a minimap2
  ```
  # simulate reads
  ```
  simulator.py genome -dna_type linear -rg $ref  -o $prefix  -n $num_reads
  ```

- MetaQUAST v5.1.0rc1
  ```
  metaquast.py -r $ref --min-contig 500 -o out --unique-mapping $assembly
  ```

- Spoa v3.4.0
  ```
  spoa -l 0 $fa
  ```

- LoRMA v0.5
  ```
  LoRMA -discarded reads.discarded.fa -output reads.corrected.fa -reads $raw_reads
  ```
