# Supplementary material for: Strainline: full-length de novo viral haplotype reconstruction from noisy long reads
Source: Genome Biol. 2022 Jan 20;23:29. doi: 10.1186/s13059-021-02587-6 (PMC8771625; doi:10.1186/s13059-021-02587-6)
Supplement: Supplementary file 2 — Additional file 2 Review history. [file 13059_2021_2587_MOESM2_ESM.docx]

Review History

**First round of review**

**Reviewer 1**

**Were you able to assess all statistics in the manuscript, including the appropriateness of statistical tests used?** Yes.

**Were you able to directly test the methods?** Yes.

**Comments to author:**

\## Summary
The authors present Strainline, a novel approach to assemble viral haplotypes and estimate their abundances from noisy long reads without a reference genome. The method can be subdivided into three steps: first, local de Bruijn graph assembly is used to eliminate errors, and then based on overlap graphs local haplotypes are iteratively extended into full-length haplotypes (if possible). Last, a filtering is performed where haplotypes with too low divergence and abundance are removed. The method outputs reconstructed haplotypes (not necessarily full length) and their respective frequencies. The authors evaluate the method on 5 simulated data sets and 2 real data sets.

The git repository is nicely structured and documented, installation is easy and the examples work.

Accept with revision.

\## Major Comments

Benchmarking:
* How does Strainline compare to reference-based long read assembly tools which are given an assembled consensus sequence as reference? Is there really any advantage in using Strainline over those approaches?
* The error correction step should be benchmarked separately and compared to alternative methods (e.g., based on local MSA, etc.)
* How do the performance measures vary for repeated synthetic benchmarks? (maybe show boxplots instead of tables)
* p.4, l.15-55, left: It would be good to list the whole frequency spectrum that was applied in the simulated data sets, not only ranges of frequency.
* p.4, l. 29, left: Typically viral populations are composed of a few very frequent strains and many low-frequency strains. It would be insightful to see how Strainline performs in such a case. In particular, you could vary the relative abundances of the Poliovirus mix not only between 2% and 50% but include lower abundances, e.g. 0.1%, 0.01%.
* p.4, l. 36, Real data: The 5-strain PVY mixture should not be claimed to be real data as it is not a mixed sample a priori, but is an in silico mixture of different samples. That is, the data is real, but the mixture is not real in a biological sense.

Methods:
* p.9, l.41, right: How would the user choose the upper limit k? How was k chosen in your applications (simulated and real data)? How does the choice of k influence results?
* p.10, l.8 left: Algorithm 1: How is the minimum cluster size m set? How was m chosen in your applications (simulated and real data)? How does the choice of m influence results?

Figures S1, S2: Why does Strainline have a measure of 0 everywhere? This seems to be an error? Obviously, the true performance cannot be assessed.


\## Minor Comments

p.1, ll. 22-24: "To accurately account for these issues, accurate reconstruction of strain-resolved genomes is a prerequisite." -- Is this really the case? This is a strong statement. Many of the aforementioned issues can already be addressed by only looking at SNVs or local haplotypes.

p.1, ll. 49-51: "very often, new outbreaks of known viruses are caused by virus variants that significantly deviate from curated reference sequence" -- Is this obvious or is there a reference one could cite?

p.2, l. 43: "offer rescue" -> "offers rescue"

p.2, ll. 8-10: "all of the assemblers presented in the literature so far fall under the category 'generic (or consensus) assembler'." -- This refers to de novo methods only, right?

p.4, left side: Each reference genome should be identified by its accession number.
p.4, l. 61-62, left: typo "This dataset is consist of five strains Potato virus Y (PVY).
" -> "This dataset consists of five Potato virus Y (PVY) strains" ?
p.4, ll. 12-13, right: "then mixed them together to generate a pseudo virus mixture" -- Does this simply mean that different fractions of reads were sampled from each strain?
p.4, ll. 34-38, right: there is something wrong with this sentence: "we subsequently excluded metaFlye, because it failed to perform the assemblies. failed to run metaFlye on our datasets"

p.5, l.37, left: How is, e.g., recall computed for the real datasets?
p.5, l.58, left: "i \in [k]" -- Is this notation well known?
p.5, ll. 35-36, right: "the content of regions shared by dif- ferent strains" -- What exactly does this mean?

p.6, l.20, right: close bracket "(because [...] assemblers, so" -> "(because [...] assemblers), so"

p.7, l.8-9, right: "easily exceed" -> "easily exceeds"

p.8, l.41-45, left: "Strainline is the only approach available that does not only assemble the viral genomes, but also estimates the abundances of the haplotypes that make part of the mix of viral strains." -- For long read, de-novo datasets.

p.9, l.11: Local de Bruijn graphs are crucial to the error correction step and are referenced with [23]. However, a brief description would make this section better understandable. How much of this is novel work, and how much is already included in d'accord?
p.9, l.25, left: What is a "significant local alignment"?
p.9, l.26-38, left: It is not clear that "Read alignment pile" (l.26), "Windows" (l.30), "Window consensus" (l.38) are referring to the components of figure 2.
p.9, l. 26, left: What is a "target read"?
p.9, l. 36: How do different window sizes affect the error correction?
p.9, ll.37-38, left: "windows may share a small overlapping interval, as one can see in "Window consensus"" -- Where can this be seen in the Windows subfigure of figure 2? The displayed windows do not seem to overlap.
p.9, l. 36, left: There is no section "DBGs for all windows"
p.9, l.42, left: The structure of the graph of DBGs is determined by the step size of windows. What is this parameter? If this step size is too big, the resulting graph will be a chain and finding the "optimal" path does not make sense.
p.9, l.42, left: What kind of scores are those?
p.9, l.47, left: How are DBGs transformed into "consensus" sequences? What is the windows consensus exactly?
p.9, l.20-51, left: Is this process (illustrated in Figure 2) done for each read?
p.9, l.12, right: "unprocessed" = not assigned to a cluster?
p.9, l. 13: "compute seed-vs- all overlaps on corrected reads using Minimap2" -- Algorithm 1 would benefit from mentioning this explicitly
p.9, l.18, right: A list of those reasonable, additional criteria to identify bad overlaps would be interesting
p.9, l.26, right: what does "in this" refer to?
p.9, l.26, right: which threshold?
p.9, l.32, right: where are those criteria listed?
p.9, ll.54-55: "it is common that the coverage for virus sequenc- ing data ultra-deep" -> "it is common that the coverage for virus sequencing data is ultra-deep"

p.10, l.51, left: "an" -> "and" ?
p.10, l.40, left: Which release/version of Spoa was used?
p.100, ll.8-9: "both of which indicates that the corresponding haplotypes reflect artifacts" -- this can be true, but might also miss low frequency/similar haplotypes.

p.11, l.19, left: Which version of PBSIM was used?
p.11, l.39, left: "supported by …" ?

Benchmarking:
* It would be good to document which parameters (k,m, maxLD, maxOH, etc.) were used for the benchmarking.

table 1-5: To better understand measures such as N50, it would be good to mention the length of each genome in the text.

supp., p.1.: The versions "snapshot ()" and "version: 0.0" should be replaced with something more meaningful.

Github: It would be nice to publish Strainline as a proper Python package on https://pypi.org/

**Reviewer 2**

**Were you able to assess all statistics in the manuscript, including the appropriateness of statistical tests used?** Yes. The statistics presented in the manuscript is generally appropriate.

**Were you able to directly test the methods?** No.

**Comments to author:**

The paper is well written, the proposed method is well described and the problem of reconstructing
intra-host viral variants from 3d generation sequencing data is very hot.

My main concern is that there is no comparison with the reference based methods, e.g. similar to one offered for SAVAGE.
For example, if one would assemble a single "best" variant, e.g. using Canu or Strainline, and then using this variant as a reference
applies reference based methods e.g. PredictHaplo or CliqueSNV - would be result superior to just Strainline? In other words,
for viruses, is the diversity high enough to assemble different variants rather than assemble a single reference and then use
existing reliable reference-based methods? Intra-host viral diversity may be high, but still usually it much less than difference between
strains and for COVID the difference is really relatively small.

What should be the level of intra-host diversity that would justify assembly of different variants? Is it true that for a population
with a major single variant of 90% frequency Strainline will output just a single variant and then using it as a reference is justifiable?

Another issue - what is the lowest detectable frequency for any particular level of detectability?
For example, is it possible to say that 10% -frequency variants can be detected with the error rate below 1%?
Or, for 1% ER Strainline needs at least 20% frequency?

Finally, it would be very useful to report how Strainline absorbs the error rate if reads, at least certain comments on the highest error rate
that can be still reasonably handled.

**Authors Response**

**Point-by-point responses to the reviewers’ comments:**

Reviewer #1: ## Summary

The authors present Strainline, a novel approach to assemble viral haplotypes and estimate their abundances from noisy long reads without a reference genome. The method can be subdivided into three steps: first, local de Bruijn graph assembly is used to eliminate errors, and then based on overlap graphs local haplotypes are iteratively extended into full-length haplotypes (if possible). Last, a filtering is performed where haplotypes with too low divergence and abundance are removed. The method outputs reconstructed haplotypes (not necessarily full length) and their respective frequencies. The authors evaluate the method on 5 simulated data sets and 2 real data sets.

The git repository is nicely structured and documented, installation is easy and the examples work.

Accept with revision.

\## Major Comments

Benchmarking:

* How does Strainline compare to reference-based long read assembly tools which are given an assembled consensus sequence as reference? Is there really any advantage in using Strainline over those approaches?

*RSP.: In agreement with the suggestions from Reviewer #2, we have added PredictHaplo or CliqueSNV in the benchmarking experiments. However, PredictHaplo failed to run on the long-read datasets used, due to an existing issue. We reported this in the PredictHaplo github repository  (https://github.com/cbg-ethz/PredictHaplo/issues/1), but the issue has not been resolved yet.*

*For all simulated PacBio read virus mixtures (note that CliqueSNV is rather tailored to PacBio, not ONT), CliqueSNV fails to reconstruct the majority of haplotypes from virus mixtures (Haplotype coverage: 10%~77%) no matter using the high quality (highest abundant strain) reference or using an ad hoc, bootstrap consensus sequence generated by Wtdbg2 or Strainline as the reference. We performed these experiments in exchange with the authors of CliqueSNV.*

*The reason apparently is the great number of SNPs that distinguish the haplotype, because the great number implies an excessive amount of cliques to be computed. As discussed with the authors, there is no sufficiently fast solution for this problem at this point in time.*

*Please see Table 2, Table S2 and Results section for details on the new experiments. In summary, Strainline has substantial advantages over these approaches.*

* The error correction step should be benchmarked separately and compared to alternative methods (e.g., based on local MSA, etc.)

*RSP.: In addition to benchmarking analysis for assemblies, we now also benchmarked several error correction tools, among which Daccord (integrated in Strainline) and other widely used tools, such as Canu (correction module), Racon, LoRMA, and CONSENT. Canu and Racon are based on multiple sequence alignments (MSAs), whereas LoRMA and CONSENT are based on both de Bruijn graphs and MSAs.*

*Racon and CONSENT failed to handle the virus data sets, apparently due to the high sequencing coverage (20,000x); note that Racon and CONSENT are generic error correction tools, so not tailored towards virus sequencing datasets. The explanation is probably the massive amount of read overlaps that arises in deep sequencing, which consumes excessive amounts of computational resources during the downstream analysis (program crashed on a machine with 48 cores and 500GB RAM).*

*The benchmarking results for error correction (with Canu, LoRMA and Racon remaining) are shown in Tables S4 (PacBio CLR) and S5 (ONT). The results show that Daccord achieves 3~65 times lower error rate on PacBio CLR data, and 2~5 times lower error rate on ONT data, in comparison to Canu and LoRMA. All three tools keep nearly 100% haplotype coverage (so do not lose genome information) and show comparable read length (N50). This justifies using Racon as a preferred choice for correcting errors in virus genome long reads.*

*Please see also the subsection “Error correction evaluation” in the Results section for the corresponding discussion.*

* How do the performance measures vary for repeated synthetic benchmarks? (maybe show boxplots instead of tables)

*RSP.: We simulated additional five “6-strain poliovirus mixture” data sets for both PacBio CLR and ONT reads, as a representative type of dataset, to evaluate the robustness of tools with respect to random effects induced by the simulation procedure.*

*Results are shown, now as boxplots (we thank for the suggestion, agreeing that this yields a clearer presentation), in Figure S4. In summary, Strainline is very robust with respect to possible simulation random effects, and outperforms Canu and Wtdbg2 in terms of robustness of the main assembly metrics, such as haplotype coverage, error rate and N50.*

*Please see the subsection “Robustness evaluation” in the Results section for a discussion.*

* p.4, l.15-55, left: It would be good to list the whole frequency spectrum that was applied in the simulated data sets, not only ranges of frequency.

*RSP.: We have added a new table (Table 1) for an improved description of the data. In this table, the whole frequency spectrum is listed as well.*

* p.4, l. 29, left: Typically viral populations are composed of a few very frequent strains and many low-frequency strains. It would be insightful to see how Strainline performs in such a case. In particular, you could vary the relative abundances of the Poliovirus mix not only between 2% and 50% but include lower abundances, e.g. 0.1%, 0.01%.

*RSP.: We have simulated two new 6-strain poliovirus mixtures, called “6-strain poliovirus (la1) mixture” and “6-strain poliovirus (la2) mixture” (see Table1 for details; ‘la’ = low abundance). These contain lower abundant strains as suggested, e.g. 0.1% (la1) or 0.01% (la2), respectively.*

*In addition, we also added a new 5-strain SARS-CoV-2 mixture, called “5-strain SARS-CoV-2 (la) mixture” , which contains lower abundant strains, e.g. of relative frequencies 0.1%, 1%.*

*Benchmarking results for these three new data sets are also shown in Table S3.*

*As a brief summary, Strainline can reconstruct haplotypes of only approximately 0.1%, but no longer of 0.01%. This, however, has no methodological reasons: the coverage of strains of 0.01% in a 20,000x dataset amounts to 2x. Clearly, 2x coverage of a strain results in plenty of coverage breaks, which prevents the assembly of such strains just from the point of view of the data. No method can deal with that.*

*Reconstructing strains of 0.01% therefore would require coverage being larger on at least one order of magnitude (e.g. 200,000x), which exceeds current standards.*

*In the following, for convenience, we cite the description of the new experiments as included in the Results section.*

*“Strainline yields near-perfect assemblies on six out of eight datasets (HIV, Poliovirus, Poliovirus (la1), HCV, ZIKV and SARS-CoV-2). That is, all ground truth haplotypes in the mixed samples are reconstructed to their full extent (HC~100%, recall=100%), at very low error rates (0.002% ~0.3%) and without misassemblies. It also obtains the exact number of true haplotypes on four datasets (precision is 100%), and overestimates the number of haplotypes in the 6-strain Poliovirus (la1) mixture (precision=75%, recall=100%) and 5-strain SARS-CoV-2 mixture (precision=71.4%,  recall=100%). Strainline fails to reconstruct all haplotypes on two datasets, namely, 6-strain Poliovirus (la2) (HC=65.3%) and  5-strain SARS-CoV-2 (la) (HC=60.8%). We recall that these two datasets are particularly challenging because they  contain strains of very low abundance (0.01% , 0.1%) and divergence (0.2%, 0.3%); see Table 1 for details. Of note, while Strainline still accurately reconstructs Poliovirus strains of 0.1% (amounting to 20x coverage), Strainline struggles to do that for SARS-CoV-2 strains. The explanation for this effect is the fact that the SARS-CoV-2 genome is 4 times longer than the Poliovirus genome (~30000bp versus ~7500bp). The expected amount of coverage breaks for a 20x covered strain in a 30000bp genome is too large to still allow for accurate full-length reconstruction, while still being feasible for genomes of 7500 bp in length.”*

* p.4, l. 36, Real data: The 5-strain PVY mixture should not be claimed to be real data as it is not a mixed sample a priori, but is an in silico mixture of different samples. That is, the data is real, but the mixture is not real in a biological sense.

*RSP.: We labeled 5-strain PVY as a “mock community” in Table 1, and now call it “experimental” rather than “real” data.*

Methods:

* p.9, l.41, right: How would the user choose the upper limit k? How was k chosen in your applications (simulated and real data)? How does the choice of k influence results?

*RSP.: In general, we recommend users to choose k between 50~200, where k=100 is a typical choice. Accordingly, we chose k=100 in most of our applications. Full details on choices of parameters used in our experiments are published in Code Ocean: https://codeocean.com/capsule/5344636/tree/v1*

*We investigated the influences of varying k in Algorithm 1. The results are shown in Table S8. It shows that the assembly performance is robust when k ranges between 60~200.*

*Note that using larger k does not necessarily improve assembly performance, while, however, requiring more computational resources.*

*We have added a new subsection “Parameter settings” in the Methods section, where we provide corresponding descriptions.*

* p.10, l.8 left: Algorithm 1: How is the minimum cluster size m set? How was m chosen in your applications (simulated and real data)? How does the choice of m influence results?

*RSP.: We used m=1 in all applications, which means that varying m was not an option by design anyway. Accordingly, we removed everything relating to m from Algorithm 1.*

*Figures S1, S2: Why does Strainline have a measure of 0 everywhere? This seems to be an error? Obviously, the true performance cannot be assessed.

*RSP.: Thank you for pointing this out. This has been an error indeed. We have corrected this now.*

\## Minor Comments

p.1, ll. 22-24: "To accurately account for these issues, accurate reconstruction of strain-resolved genomes is a prerequisite." -- Is this really the case? This is a strong statement. Many of the aforementioned issues can already be addressed by only looking at SNVs or local haplotypes.

*RSP.: We agree that the statement is too strong. Still, we feel that long range reconstruction can at least be helpful to have a clear view of effects, even if this is not an immediate necessity (we hope that one can agree on that?).*

*Accordingly, we now say: “To accurately account for these issues, accurate reconstruction of strain-resolved genomes can be very helpful, if not even necessary in some cases.”*

p.1, ll. 49-51: "very often, new outbreaks of known viruses are caused by virus variants that significantly deviate from curated reference sequence" -- Is this obvious or is there a reference one could cite?

*RSP.: We have inserted two citations in the manuscript (see here and here).*

p.2, l. 43: "offer rescue" -> "offers rescue"

*RSP.: We have corrected this as suggested.*

p.2, ll. 8-10: "all of the assemblers presented in the literature so far fall under the category 'generic (or consensus) assembler'." -- This refers to de novo methods only, right?

*RSP.: Yes. We have adjusted the sentence.*

p.4, left side: Each reference genome should be identified by its accession number.

*RSP.: We have provided a new table (Table S1) to display accession numbers of genomes used in the manuscript.*

p.4, l. 61-62, left: typo "This dataset is consist of five strains Potato virus Y (PVY).

" -> "This dataset consists of five Potato virus Y (PVY) strains" ?

*RSP.: We have corrected this as suggested.*

p.4, ll. 12-13, right: "then mixed them together to generate a pseudo virus mixture" -- Does this simply mean that different fractions of reads were sampled from each strain?

*RSP.: We did not perform subsampling. Instead, we mixed all the original reads of each strain, because each strain has different coverage. This makes for a mixture of strains of variable abundances.  (see Table1).*

p.4, ll. 34-38, right: there is something wrong with this sentence: "we subsequently excluded metaFlye, because it failed to perform the assemblies. failed to run metaFlye on our datasets"

*RSP.: We have corrected this.*

p.5, l.37, left: How is, e.g., recall computed for the real datasets?

*RSP.: The computation is the same as for the simulated datasets because we know the true genome of each strain in the experimental 5-strain PVY mixture (which we now correctly refer to as mock community, which may resolve any possible confusion).*

*For the real SARS-CoV-2 sample, we did not compute measures such as recall as we do not know the ground truth.*

p.5, l.58, left: "i \in [k]" -- Is this notation well known?

*RSP.: Not sure how well known it is outside of mathematics, but it seems to be a common shorthand for {1,...,k} in mathematics. We have replaced it with the explicit {1,...,k} to avoid confusion.*

p.5, ll. 35-36, right: "the content of regions shared by different strains" -- What exactly does this mean?

*RSP.: With this, we tried to express that two different strains can share identical sequence. Chances for this effect increase on genomes being longer.*

p.6, l.20, right: close bracket "(because [...] assemblers, so" -> "(because [...] assemblers), so"

*RSP.: We have corrected this as suggested.*

p.7, l.8-9, right: "easily exceed" -> "easily exceeds"

*RSP.: We have corrected this.*

p.8, l.41-45, left: "Strainline is the only approach available that does not only assemble the viral genomes, but also estimates the abundances of the haplotypes that make part of the mix of viral strains." -- For long read, de-novo datasets.

*RSP.: We have changed as “Strainline is the only long-read de novo assembly approach...”*

p.9, l.11: Local de Bruijn graphs are crucial to the error correction step and are referenced with [23]. However, a brief description would make this section better understandable. How much of this is novel work, and how much is already included in d'accord?

*RSP.: We have briefly explained how to apply the local de Bruijn graph for error correction in the 3rd, 4th and 5th paragraph of subsection “Correcting sequencing errors” in the Methods section. A  corresponding schematic diagram can also be seen in Figure 2.*

*As for usage of Daccord, we have integrated it as is into our workflow, while of course adjusting parameters that specify important quantities referring to data sets, as required. The justification is that Daccord works just fine, without any further additions or modification, for virus deep sequencing data sets. Note another time that this came as a surprise, because it does not reflect the intended use case of the authors.*

*To clarify this, we have added the following text in the subsection “Correcting sequencing errors”: “We integrate Daccord, as originally suggested [27[, by straightforwardly adjusting parameters so as to account for particularities of data sets under consideration here, but without any methodical or theoretical adjustments, into our workflow.”*

p.9, l.25, left: What is a "significant local alignment"?

*RSP.: We were meaning ‘local alignment of high confidence’. We have corrected this in the manuscript.*

p.9, l.26-38, left: It is not clear that "Read alignment pile" (l.26), "Windows" (l.30), "Window consensus" (l.38) are referring to the components of figure 2.

*RSP.: To enhance understanding, we have now added additional information in the corresponding paragraph to immediately make sure that these terms refer to components of Figure 2..*

p.9, l. 26, left: What is a "target read"?

*RSP.: The target read is the read to be corrected. We have added this explanation in the manuscript.*

p.9, l. 36: How do different window sizes affect the error correction?

*RSP.: We tested the performance of Strainline's error correction step (which reflects using Daccord) using window sizes ranging from 20 to 200 (see Figure S5). These experiments show that optimal performance is achieved for windows of 30 to 50bp. Larger window sizes require more computational resources.*

*In agreement with these experiments, we determined a window size of 40 bp as a reasonable default.  Please see the subsection “Error correction evaluation” in the Results section.*

p.9, ll.37-38, left: "windows may share a small overlapping interval, as one can see in "Window consensus"" -- Where can this be seen in the Windows subfigure of figure 2? The displayed windows do not seem to overlap.

*RSP.: We have modified Figure 2, which now indicates the overlap between windows in ‘Window segmentation’. Further, in ‘Window consensus’  in Figure 2, a gray shaded area virtually connecting two graphs is to indicate the overlap of the two graphs.*

p.9, l. 36, left: There is no section "DBGs for all windows"

*RSP.: There is now a text passage that explicitly explains “DBGs for all windows”, which is an element of Figure 2.*

p.9, l.42, left: The structure of the graph of DBGs is determined by the step size of windows. What is this parameter? If this step size is too big, the resulting graph will be a chain and finding the "optimal" path does not make sense.

*RSP.: In all our experiments, the step size is set as 10bp, which is controlled by the parameter -a: advance size| in Daccord.*

*We have added the following text at the corresponding location in the manuscript: “Importantly, windows share an overlapping interval of 30bp (that is, the step size of a window is 10bp, which is controlled by the parameter -a: advance size in Daccord)...”*

p.9, l.42, left: What kind of scores are those?

*RSP.: We have explained the scores in more detail in the manuscript.*

p.9, l.47, left: How are DBGs transformed into "consensus" sequences? What is the windows consensus exactly?

*RSP.: Now we have explained what window consensus is and how DBGs are transformed into consensus sequences in the 4th and 5th paragraph of subsection “Correcting sequencing errors” of the Methods section.*

*To clarify this further, we have also added the following text to the caption of Figure 2: “Window consensus refers to the highest scoring sequences (see main text for explanations) through the DBGs of small windows over the read alignment pile.”*

p.9, l.20-51, left: Is this process (illustrated in Figure 2) done for each read?

*RSP.: Yes. We have added an explanation in the caption of Figure 2.*

p.9, l.12, right: "unprocessed" = not assigned to a cluster?

*RSP.: Yes. We have fixed this in the manuscript.*

p.9, l. 13: "compute seed-vs- all overlaps on corrected reads using Minimap2" -- Algorithm 1 would benefit from mentioning this explicitly.

*RSP.: We now mention this explicitly in Algorithm 1 as suggested.*

p.9, l.18, right: A list of those reasonable, additional criteria to identify bad overlaps would be interesting.

*RSP.: We now have provided clearer explanations about those reasonable, additional criteria in “Read cluster generation” in the Methods section:*

*“Bad overlaps are filtered out according to reasonable, additional criteria, such as minimum read overlap length (--minOvlpLen), minimum level of sequence identity for read overlaps (--minIdentity), and in case of self-overlaps, duplicates or internal matches (maximum overhang length of overlaps, --maxOH). Overlaps that do not satisfy these criteria are removed.”*

p.9, l.26, right: what does "in this" refer to?

*RSP.: We have removed this ambiguous text “in this”, and replaced it as “To define these criteria concretely”.*

p.9, l.26, right: which threshold?

*RSP.: For better understanding, we have changed the original text as “Enforced by choosing relatively strict thresholds for these criteria (see [39] for the details)...”*

*For example: we used thresholds: --minOvlpLen 1000, --minIdentity 0.99,  --maxOH 50, etc.*

*Full details on choices of parameters used in our experiments are published in Code Ocean: https://codeocean.com/capsule/5344636/tree/v1*

p.9, l.32, right: where are those criteria listed?

*RSP.: In consideration of the above 4 comments overall, we have reformulated these corresponding two paragraphs and added some more details in the manuscript.*

*So, “those criteria listed” are mentioned above (see p.9, I.18), which should be also clear now.*

p.9, ll.54-55: "it is common that the coverage for virus sequenc- ing data ultra-deep" -> "it is common that the coverage for virus sequencing data is ultra-deep"

*RSP.: We have corrected this.*

p.10, l.51, left: "an" -> "and" ?

*RSP.: We have corrected this.*

p.10, l.40, left: Which release/version of Spoa was used?

*RSP.: Spoa v3.4.0. We have added this to the supplement.*

p.100, ll.8-9: "both of which indicates that the corresponding haplotypes reflect artifacts" -- this can be true, but might also miss low frequency/similar haplotypes.

*RSP.: We changed as “...are likely to  reflect artifacts”.*

p.11, l.19, left: Which version of PBSIM was used?

*RSP.: We used PBSIM v1.0.3-h2d504032. Now we have added the commands and versions of tools used in the manuscript to the supplement.*

p.11, l.39, left: "supported by …" ?

*RSP.: We have reformulated this.*

Benchmarking:

* It would be good to document which parameters (k,m, maxLD, maxOH, etc.) were used for the benchmarking.

*RSP.: (See also an earlier response) We set m=1 (which means that we removed this parameter, as we do not make use of it). In general we operted with k=100, maxLD=0.01, maxOH=50 in most of our applications. For full details on choices of parameters used in our benchmarking experiments, see Code Ocean: https://codeocean.com/capsule/5344636/tree/v1*

*We have also added a new subsection “Parameter settings” in the Methods section.*

table 1-5: To better understand measures such as N50, it would be good to mention the length of each genome in the text.

*RSP.: A new table (Table1) now shows detailed descriptions of the benchmarking data sets, including the length of the underlying genomes.*

supp., p.1.: The versions "snapshot ()" and "version: 0.0" should be replaced with something more meaningful.

*RSP.: Now we have added a new section “Commands and versions of tools used for comparison” in the supplement.*

Github: It would be nice to publish Strainline as a proper Python package on https://pypi.org/

*RSP.: We agree and thank for the recommendation. We are planning to do this in the future.*

Reviewer #2: The paper is well written, the proposed method is well described and the problem of reconstructing  intra-host viral variants  from 3d generation sequencing data is very hot.

My main concern is that there is no comparison with the reference based  methods, e.g. similar to one offered for SAVAGE. For example, if one would assemble a single  "best" variant, e.g. using Canu or Strainline, and then using this variant as a reference applies reference based methods e.g. PredictHaplo or CliqueSNV - would be result superior to just Strainline? In other words, for viruses, is the diversity high enough to assemble different variants rather than assemble a single reference and then use existing reliable reference-based methods? Intra-host viral diversity may be high, but still usually it much less than *difference between strains and for COVID the difference is really relatively small.*

*RSP.: We thank you for making this point. Please note that the comment is in agreement with one of the requests of Reviewer #1.*

*To address this point, we considered PredictHaplo and CliqueSNV in the benchmarking experiments, as suggested (we agree on this choice reflecting exemplary, popular and/or most recent approaches). However, we failed to run PredictHaplo on the long read data sets under consideration here. The reason is a so far unresolved issue that we reported in the github repository (https://github.com/cbg-ethz/PredictHaplo/issues/1).*

*Further, we ran CliqueSNV, which is tailored mostly to processing PacBio data, on all simulated PacBio datasets under consideration here. Results show that CliqueSNV does not reconstruct the majority of haplotypes from the corresponding virus mixtures (Haplotype coverage: 10%~77%). In this, the particular choice of reference genome does not have much of an influence, where we consider the high quality (highest abundant strain) reference or ad hoc bootstrap consensus sequence generated by Wtdbg2 or Strainline as possible choices for  references.*

*We performed these experiments in personal communication exchange with the authors of CliqueSNV, so as to ensure optimal choices of parameters etc for CliqueSNV. The reason for the difficulties that CliqueSNV experiences apparently is the great number of SNPs that distinguish the haplotypes, which implies an excessive amount of cliques to be computed. As discussed with the authors, there is no sufficiently fast solution for this problem at this point in time.*

*Please see Table 2, Table S2 and Results section for details on the new experiments. In summary, Strainline has substantial advantages over these approaches.*

*In addition, as implemented 1-to-1 according to the suggestions from above, we also tested “Strainline+CliqueSNV” (using Strainline for generation of reference, applied by CliqueSNV) named as “CliqueSNV-b-ref-corr (Strainline) in Table S3”) on two low-frequent strain datasets, namely, 6-strain poliovirus (la2) mixture and 5-strain SARS-CoV-2 (la) mixture, for which Strainline failed to reconstruct all haplotypes in the mixture. However, the strategy  “Strainline+CliqueSNV” did not improve results. In fact, the strategy yields only one haplotype as output, which is (clearly) inferior to Strainline. We recall that the lowest genome divergence of these two datasets amounts to 0.2% and 0.3%, respectively, which is very challenging. Please see Table S3 for the details.*

What should be the level of intra-host diversity that would justify assembly of different variants? Is it true that for a population with a major single variant  of 90% frequency Strainline will output just a single variant and then using it as a reference is justifiable?

*RSP.: To provide detailed answers to this question, we performed new experiments using mixtures of two strains of varying ratios of abundance and divergence (using simulated PacBio CLR reads), to determine the limits of Strainline with respect to differences of strains both in terms of ratio of abundances and divergence.*

*Results are shown in (the new) Figure 3. It shows that Strainline can reconstruct all the haplotypes for abundance ratios as low as 1:100, if divergence is at least 0.5%. Please see the new subsection “Effect of divergence-vs-ratio” in the Results section.*

*Given the results from earlier experiments, it is very questionable that any reference-based strategy can match these performance rates given the methods that are currently available. Note that reference-based methods are not necessarily simpler in terms of methodology. The crucial difficulty for reference-based methods is to distinguish true variants from errors relative to the reference, where the reference may introduce biases due to the variants it harbors in its own right.*

*Based on the results reported here, and also reported in earlier work for short read data, we are convinced that reference-based methods experience certain limits that are hard (impossible?) to overcome. While, of course, we cannot exclude that some future work may overcome them.*

Another issue - what is the lowest detectable frequency for any particular level of detectability?

For example, is it possible to say that 10% -frequency variants can be detected with the error rate below 1%? Or, for 1% ER Strainline needs at least 20% frequency?

*RSP.: As already pointed out, we have performed new experiments using two-strain mixtures with varying ratios of abundance (1:1000 to 1:1) and divergence, see above.*

*The clear limit for detectable frequency, to the best of our understanding, is not so much the relative frequency in comparison with the error rate, although this also plays a role. However, relative frequency alone in relation to coverage overall makes for a clear limit: if coverage of a single strain drops below, say, 5X or 10X (which also depends on the length of the genome of the virus), strains can no longer be reconstructed because of the coverage breaks that affect the strains. This plays a role for both reference-based and de novo tools, because coverage breaks relate to crucial pieces of data just missing.*

Finally, it would be very useful to report how Strainline absorbs the error rate if reads, at least certain comments on the highest error rate that can be still reasonably handled.

*RSP.: To investigate the effect of sequencing error rate on viral quasispecies assembly, we simulated both PacBio CLR and ONT reads with different sequencing error rates (ranging from 5% to 30%). Assembly results are shown in Supplementary Table S9 (CLR) and Table S10 (ONT).*

*One observes that Strainline successfully reconstructs all true haplotypes at nearly perfect coverage (HC: ~100%) and correctly estimates the number of haplotypes in all PacBio CLR data sets regardless of the particular sequencing error rate (5%~30%). Likewise, Strainline shows very similar performances on ONT data sets with the only exception that Strainline experiences difficulties on ONT data for sequencing error rates of 30%; note that this is due to the error correction module as implemented using Daccord, which shows better results for CLR than for ONT data.*

**Second round of review**

**Reviewer 1**

1) Thank you for adding CliqueSNV to the benchmark. It does now indeed look like Strainline substantially outperforms this approach. It is unfortunate that PredictHaplo was not able to produce results as its probabilistic approach could have been able to deal with the issues leading to clique enumeration problems in CliqueSNV.

2) In the section "Error correction evaluation" the authors only compare Daccord to other tools but isn't Strainline doing more error correction than Daccord (as described in Figure 2)? Or is Daccord doing everything that is illustrated in Figure 2? Please, explain more explicitly the differences to Daccord with respect to the error correction in strainline.
The authors write "(with Canu, LoRMA and Racon remaining)" but then continue with "The results show that Daccord achieves". Tables S4 and S5 also mention Daccord and not Racon. In the end the authors however write again "This justifies using Racon". Whenever the authors say Racon do they actually mean Daccord (this seems to fit the added discussion in the paper)?

3) The authors state "Strainline outperforms Canu". This does not seem obvious in figure S4 (B) for ONT reads. Can the authors comment on why canu is seemingly performing better than strainline?


Minor comments

* Figure 3 - description: typos: "srelative" ?, "divergencetrain" ?, "the ratio ofpresents the"? , "refer to shows" ?, "DThe darkereeper colors indicate better performance.the blue, the better the assembly performance."

* p.11, l.37, right: "datas ets" → "datasets" ?

* p.14, l.23. left: Results are not in Table S6 but in Table S8

* Figure S1 (d,g,h), figure S2 (a): not all methods mentioned in the legend appear in all subplots. What is the reason for this? If lines are overlapping the visualization should be improved.

**Authors Response**

**Point-by-point responses to the reviewers’ comments:**

Reviewer #1: 1) Thank you for adding CliqueSNV to the benchmark. It does now indeed look like Strainline substantially outperforms this approach. It is unfortunate that PredictHaplo was not able to produce results as its probabilistic approach could have been able to deal with the issues leading to clique enumeration problems in CliqueSNV.

We appreciate CliqueSNV as a very interesting approach in general. To the best of our judgment, the issues are not of conceptual nature, but are due to efficiency issues that one could possibly fix.

What we are convinced of in any case is that the biases introduced by PredictHaplo are rather non-negligible. We are speaking not just because of theoretical considerations, but because of many practical application runs that we analyzed earlier in time.

2) In the section "Error correction evaluation" the authors only compare Daccord to other tools but isn't Strainline doing more error correction than Daccord (as described in Figure 2)? Or is Daccord doing everything that is illustrated in Figure 2? Please, explain more explicitly the differences to Daccord with respect to the error correction in strainline.

The authors write "(with Canu, LoRMA and Racon remaining)" but then continue with "The results show that Daccord achieves". Tables S4 and S5 also mention Daccord and not Racon. In the end the authors however write again "This justifies using Racon". Whenever the authors say Racon do they actually mean Daccord (this seems to fit the added discussion in the paper)?

Reply: Sorry for the confusion. Daccord does everything that is illustrated in Figure 2. Please see “Correcting sequencing errors” in the “Methods” section where we have explained this: “*We integrate Daccord, as originally suggested \cite{tischler2017non}, by straightforwardly adjusting parameters so as to account for particularities of datasets under consideration here, but without any methodical or theoretical adjustments, into our workflow.* “

As for the second part, yes, it should be Daccord rather than Racon. We have resolved the issue and adapted the texts.

3) The authors state "Strainline outperforms Canu". This does not seem obvious in figure S4 (B) for ONT reads. Can the authors comment on why canu is seemingly performing better than strainline?

Reply: In Figure S4(B) for ONT reads, it shows Canu achieves lower error rate than Strainline. However, this error rate only reflects the average error over strains. For example, in one case, Canu generates 2 strains (haplotype coverage = 2/6 = 33.3%) : strain1 with 0.053% error rate , strain2 with 0% error rate.

Whereas Strainline generates 6 strains (haplotype coverage = 100%): both strain1 and strain2 ( two most frequent strains) with 0% error rate, but other strains (less frequent) with 0.05%~0.12% error rate.

Relative to this, Strainline still outperforms Canu in terms of the error rates it achieves on the most predominant (most frequent) strains. So, virtually Strainline is better, while correctly adding lower frequent strains where error rates (naturally) suffer a bit. As a consequence, when taking the average, Strainline looks somewhat worse than Canu, which as we feel distorts the right idea about error rates.

We have added the comments in the “Results: Robustness evaluation” section:

“*Note that in Figure S4 (B) for ONT reads Canu achieves lower error rate than Strainline. This is because Strainline generates almost all strains (including the less frequent strains), thus raising the average error rate, whereas Canu can only reconstruct the most abundant strains. In fact, Strainline still achieves lower error rates than Canu when directly comparing it on the most abundant strains.*”

Minor comments

* Figure 3 - description: typos: "srelative" ?, "divergencetrain" ?, "the ratio ofpresents the"? , "refer to shows" ?, "DThe darkereeper colors indicate better performance.the blue, the better the assembly performance."

Reply: We have fixed all of this.

* p.11, l.37, right: "datas ets" → "datasets" ?

Reply: Yes, we have changed it.

* p.14, l.23. left: Results are not in Table S6 but in Table S8

Reply: We have fixed this.

* Figure S1 (d,g,h), figure S2 (a): not all methods mentioned in the legend appear in all subplots. What is the reason for this? If lines are overlapping the visualization should be improved.

Reply: It is because lines are overlapping. We have improved these subplots.
